# Factors of emotional distress in lymphoma: A systematic review

**DOI:** 10.1002/cam4.6069

**Published:** 2023-05-17

**Authors:** Kai Ping Tan, Dipti Talaulikar, Brett Scholz

**Affiliations:** ^1^ School of Medicine and Psychology, College of Health and Medicine Australian National University Canberra Australia

**Keywords:** cancer, distress, lymphoma, oncology, risk factors, survivorship

## Abstract

**Objective:**

Distress is prevalent among lymphoma patients/survivors. Current processes of distress identification rely on self‐reporting by patients/survivors, which may be limited by their willingness to report symptoms. To help identify patients/survivors at greater risk, this systematic review aims to comprehensively review factors that may contribute to distress in lymphoma patients/survivors.

**Methods:**

PubMed was systematically searched for peer‐reviewed primary articles (1997–2022) consisting of standardised keywords “lymphoma” and “distress.” Information from 41 articles was integrated via narrative synthesis.

**Results:**

Consistent risk factors of distress include younger age, relapsed disease, and greater comorbidities and symptom burden. Active treatment and the transition from treatment to post‐treatment could be challenging phases. Adequate social support, adaptive adjustment to cancer, engaging in work and healthcare professionals' support may mitigate distress. There is some evidence that older age may be associated with greater depression and life changes/experiences may shape how individuals cope with lymphoma. Gender and marital status were not robust predictors of distress. Other clinical, psychological and socioeconomic factors are understudied or have mixed findings.

**Conclusions:**

While several factors of distress align with that of other cancers, more research is needed to identify significant factors of distress in lymphoma patients/survivors. The identified factors may support clinicians in identifying distressed lymphoma patients/survivors and providing interventions where necessary. The review also highlights avenues for future research and a need to routinely collect data on distress and its factors in registries.

## 1 BACKGROUND

It is estimated 1 in 40 Australians will be diagnosed with lymphoma by age 85. The Australian Institute of Health and Welfare (AIHW) estimates 7397 cases of lymphoma will be newly diagnosed in 2022.^1^ Lymphoma encompasses a heterogenous group of cancers of the lymphatic system which vary significantly in prognosis and treatment.^2^ The recent World Health Organization (WHO) classification has identified more than 80 subtypes of lymphoma^3^ ranging from indolent (i.e. slow‐growing) lymphoma which are generally incurable, to aggressive (i.e. rapidly growing) lymphoma which often respond well to treatment.^4^, ^5^ Lymphomas are broadly categorised into two groups: Hodgkin Lymphoma (HL) and Non‐Hodgkin Lymphoma (NHL).^2^ HL is less common but highly treatable, with a 5‐year survival rate of 87%.^6^ On the contrary, NHL remains the 6th most prevalent cancer in Australia and advances in treatment, particularly the introduction of monoclonal antibodies in the late 1990s, improved its 5‐year survival rate to 71%.^1^, ^6^

The experience of lymphoma from symptoms to the diagnostic process, treatment and posttreatment can be a distressing experience for many patients. Common symptoms of lymphoma include B‐symptoms (i.e. unexplained fevers, weight loss and night sweats), swollen lymph nodes in neck, armpit, or groin, persistent fatigue, chills, shortness of breath and/or itchy skin.^2^ Diagnosis and staging of lymphoma may involve several tests including PET (positron emission tomography) scan and CT (computerised tomography) scan, and bone marrow biopsy; and lymphoma treatments can range from watchful waiting to intensive treatments including chemotherapy, radiation therapy, novel treatments and/or stem cell transplant.^2^ Indeed, psychological distress is recognised as a prevalent issue among cancer patients/survivors^7^ and emerging research suggests that lymphoma patients/survivors can have significantly impaired psychological wellbeing and health‐related quality of life (HRQOL).^8^, ^9^, ^10^, ^11^, ^12^, ^13^

To address distress in cancer populations, the current dominant approach is to screen and refer patients with clinically significant distress.^14^ Distress in this context is defined as ‘a multifactorial unpleasant emotional experience of a psychological (cognitive, behavioural, emotional), social, and/or spiritual nature that may interfere with the ability to cope effectively with cancer, its physical symptoms and its treatment. Distress extends along a continuum, ranging from common normal feelings of vulnerability, sadness and fears to problems that can become disabling, such as depression, anxiety, panic, social isolation and existential and spiritual crisis’. [7, p. MS‐4]. There is some evidence that distress screening accompanied by needs assessment may be useful in improving patient–clinician communication.^15^ However, a recent commentary on this screening and referral process highlighted a lack of improvement in patient distress.^14^

Currently, little is known about the psychosocial experience of lymphoma and healthcare professionals depend primarily on patients' self‐reports of distress (e.g. distress screening and patients asking for help) [e.g.^16^], which may be confounded by patients' willingness to report distress. Reviews suggest that many patients develop short‐term symptoms but some patients/survivors continue to develop severe symptoms over time.^12^, ^13^ This highlights the need to identify risk factors of distress in this population. This could inform healthcare professionals on who for, where and when might psychosocial services be most necessary, as well as provide some guidance on modifiable factors that may attenuate distress.

Existing systematic reviews have mainly synthesised studies of HRQOL in this population.^8^, ^9^, ^10^, ^11^, ^17^, ^18^ However, being one domain of a broad and multidimensional HRQOL, emotional distress is rarely discussed extensively in these reviews. This distinction between general HRQOL and emotional distress is important because several reviews have suggested differential influence of these factors on the domains of HRQOL. For example, one of the findings from Leak and colleagues' review^8^ was that older NHL survivors had worse physical but better mental QOL compared to their younger counterparts. Of the reviews that did discuss factors of distress, conclusions are often drawn across a limited number of studies, ranging from 1 to 3 studies per factor.^10^, ^12^, ^13^ Although risk factors of distress have been identified for various cancers more broadly,^19^, ^20^ to the authors' knowledge, no review has specifically examined factors of distress in this population. This systematic review aims to comprehensively evaluate factors that may contribute to distress in lymphoma patients/survivors.

## 2 METHODS

Guided by the Cochrane Handbook for Systematic Reviews^21^ and the PRISMA (Preferred Reporting Items for Systematic Reviews and Meta‐Analyses) statement,^22^ a systematic review of articles examining factors of distress in lymphoma patients/survivors was conducted.

### 2.1 Search Strategy

Human studies published in English were systematically searched on PubMed on 15 April 2022. Considering the potential impact of treatment experience on QOL, studies were limited to those published from 1997. This was the year rituximab, the first monoclonal antibody which changed treatment paradigms significantly upon inclusion, was approved for use by the Food and Drug Administration (FDA).^23^ The following MeSH (Medical Subject Headings) terms were used: *Lymphoma* AND various MeSH terms for distress (i.e. *Psychological Distress OR Stress, Psychological OR Emotions OR Stress Disorders, Traumatic OR Mental Health OR Adaptation, Psychological OR Adjustment Disorders)*.

### 2.2 Inclusion and Exclusion Criteria

Original empirical studies which were peer‐reviewed and discussed distress as an outcome variable were included in the review. Articles were excluded if the study sample included patients/survivors with other types of cancer, individuals younger than 18 years old at time of study, or consisted only of caregivers or childhood survivors.

### 2.3 Selection Process

Title/abstract screening and full‐text screening were conducted by KPT. Articles were evaluated against the established inclusion/exclusion criteria. Articles that KPT were unsure about were discussed in bi‐weekly meetings with BS and DT. EndNote 20 was used to manage and organise references.

### 2.4 Data Collection and Extraction

With supervision from BS and DT, KPT was supported in extracting data independently from the included studies. Extracted information included author(s), year published, country in which study was conducted, aim, design, measures, sample characteristics, findings relevant to factors of emotional distress and the main statistical analyses associated with these findings. When an article did not directly answer the review question, findings that provided more depth to the topic were extracted.

### 2.5 Quality Assessment

Extracted data were shared and discussed with BS and DT. Each article was evaluated by the JBI Critical Appraisal Tool^24^ and was included if there were no concerns in majority of applicable questions.

### 2.6 Synthesis Method

Narrative synthesis was used due to heterogeneity in study designs. This form of analysis can allow richer discussions around the various factors examined and was regarded as more suitable for the current review.

## 3 RESULTS

**Figure 1:**
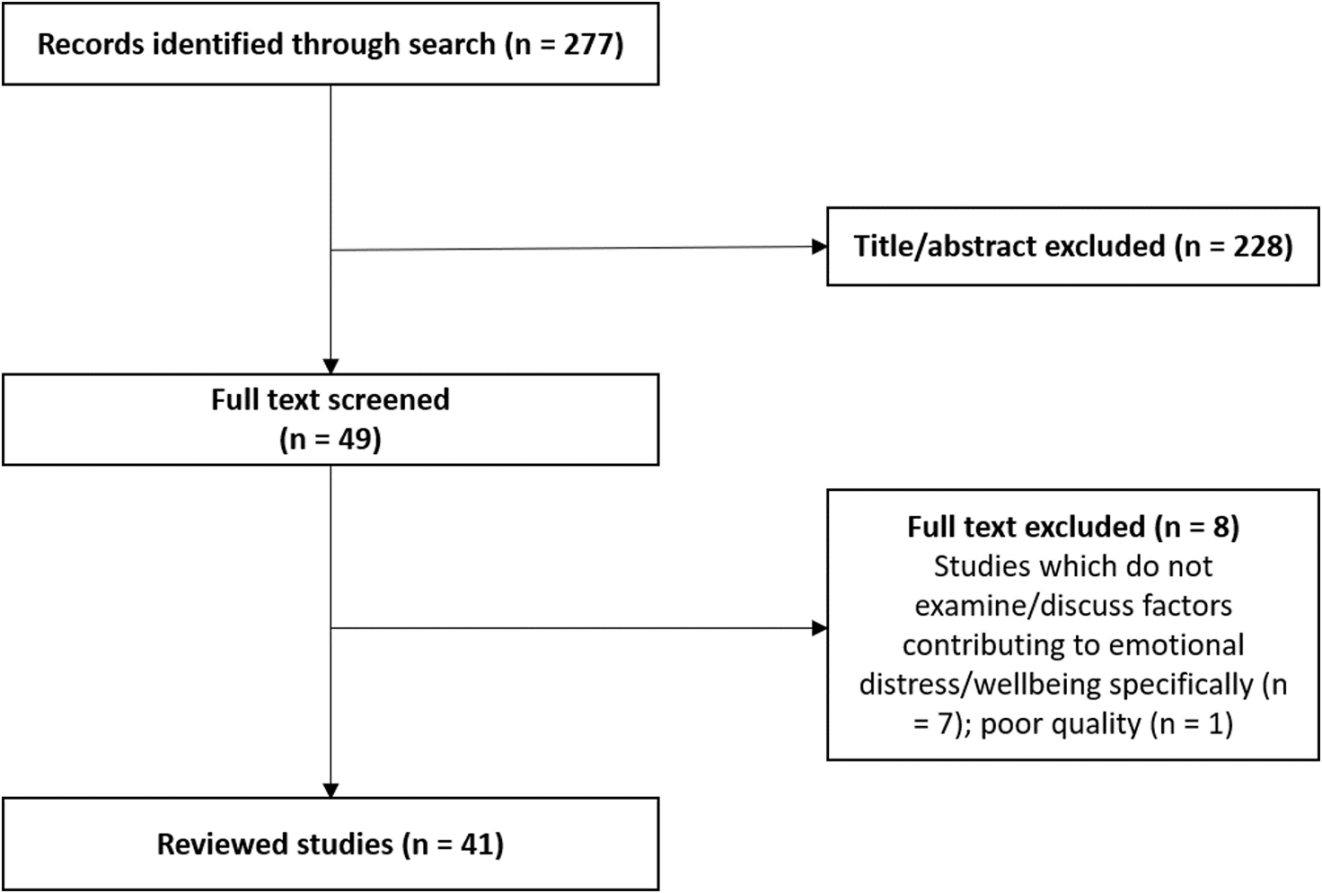
PRISMA Flowchart. Flowchart of screened articles at each stage, from number of studies at initial literature search to final reviewed studies; n = number of articles.

The search strategy yielded 277 articles. A total of 228 articles were excluded after title/abstract screening because they were irrelevant, did not examine only the population of interest, or were evaluating novel interventions or situations (e.g. COVID‐19) not within the scope of the current review. After full‐text screening, a further seven articles were excluded due to not examining emotional distress/wellbeing specifically as an outcome variable and one article was excluded due to poor quality (see Figure 1 below).

A total of 41 articles (40 studies) were included in the final analysis (for summary, see Supplementary Materials: Table 1 (quantitative studies), Table 2 (qualitative studies) and Table 3 (mixed‐methods studies)). Seventeen studies (18 articles) (43.9%) were cross‐sectional, with 7 prospective studies, 3 retrospective studies, 11 qualitative and 2 mixed‐methods. Studies were mainly conducted in United Kingdom or the United States (53.4%), with samples being predominantly non‐Hispanic white. Other countries included Australia, Brazil, China, Denmark, France, Germany, Hungary, Ireland, Israel, Norway, Spain, Sweden and the Netherlands. Across all articles, median sample size was 140, ranging from 6 participants to 8750 participants. Participant ages ranged between 15 and 92 at time of study, although some studies only reported age at diagnosis.^25^, ^26^ Three studies focused primarily on young adults,^27^, ^28^, ^29^ and three other studies had majority of participants aged ≥65.^30^, ^31^, ^32^ One qualitative study engaged in purposive sampling to ensure equal number of participants across age groups (i.e. adolescent and young adults (AYA; 18–39 years), middle age (40–65 years) and older age (> 65 years).^33^ Majority of these articles (19; 46.3%) involved all participants with lymphoma whereas some studies focussed on participants with HL (11 studies; 26.8%) or NHL (6 studies (7 articles); 17.1%), aggressive NHL (2 studies; 4.8%) or follicular lymphoma (2 studies; 4.8%). Majority of studies focused on survivors from 3 months to several decades post‐treatment (26 studies (27 articles); 63.4%).

Overall, these articles were heterogenous in their study aims. Types of distress examined included: anxiety and depression, fear of cancer recurrence, post‐traumatic stress disorder, mental health status and functioning, general distress, perceived stress and existential challenges (i.e. concerns about grief, guilt, security and identity). A variety of factors contributing to psychosocial distress were examined. These factors can be broadly grouped into four categories: sociodemographic, clinical, psychosocial and healthcare.

### 3.1 Sociodemographic Characteristics

Nine sociodemographic characteristics were discussed across included studies whereby age was most frequently examined (n = 16). Other characteristics examined are gender (n = 13), employment status (n = 6), education level (n = 8), race/ethnicity (n = 3), finance‐related factors (n = 4), marital status (n = 6), habitual status (n = 1) and whether participants had children (n = 2).

#### 3.1.1 Age

Twelve of sixteen studies suggest that age may be a significant factor,^25^, ^27^, ^31^, ^32^, ^34^, ^35^, ^36^, ^37^, ^38^, ^39^, ^40^, ^41^ although this association was not found to be significant in some studies.^26^, ^42^, ^43^, ^44^ For example, a cross‐sectional study by Smith and colleagues^32^ found that younger age at study enrolment predicts worse mental health status, even when other sociodemographic/clinical characteristics and perceived impact of cancer were accounted for. Younger age at treatment and/or survey was also associated with borderline and major anxiety, psychiatric morbidity and fear of cancer recurrence, although in this study, age was not related to depression.^40^ Among long‐term survivors, younger age at study enrolment was associated with greater PTSD symptoms^31^ and younger onset of disease was associated with experiencing more intense intrusion and avoidance symptoms.^39^ Moreover, older age was linked to lesser existential worry about cancer (i.e. being preoccupied with cancer concerns and being worried about dying from cancer or cancer coming back).^36^ Similarly, a study by Bellizzi and colleagues^37^ found that the older the participants were, the less positive and negative life changes after cancer were reported, suggesting that older individuals may be less affected by their cancer experience.

In contrast, a recent study found that HL patients aged over 30 years had significantly higher incidences of psychotropic drug (e.g. anxiolytics and antidepressants) prescriptions over 5 years compared to younger HL patients.^35^ This difference was also observed for NHL patients >70 years.^41^ While the previous findings may point to younger age being a significant risk factor for distress, it is possible that age may have differential effects depending on the type of distress examined. A prospective study by Oerlemans and colleagues^45^ highlighted that among DLBCL patients, younger age at time of first survey was associated with greater anxiety over time whereas older age at time of first survey was associated with greater depression over time. Other studies highlighted that older age (> 45–50 years) was linked with greater likelihood of depression caseness*,^25^, ^38^ whereby age remained a significant predictor of depression caseness even when other sociodemographic and clinical characteristics were accounted for.^25^ A prospective study examining young adults' changes in HRQOL post‐treatment demonstrated that depression improved over time: at 6 months, severe depression was not reported by any participants, whereas there were no significant improvements in anxiety levels.^27^ Even so, some studies did not find significant associations between age and mental health status, general distress, anxiety and/or depression.^42^, ^47^, ^48^ Moreover, age was not found to be significant predictor of actionable distress when survivorship period was accounted for^26^ or clinical levels of fear of cancer recurrence when anxiety/depression and other sociodemographic/treatment characteristics were accounted for.^43^

#### 3.1.2 Gender/Sex

Nine of thirteen studies demonstrated that gender is not a significant predictor of distress,^26^, ^42^ mental health status,^32^ fear of cancer recurrence^43^ or depression.^35^, ^38^, ^40^, ^44^, ^49^ However, there seems to be a trend for women with HL to experience greater distress. One study found that women HL and NHL long‐term survivors were more likely than men to report concerns of grief and guilt over death of other cancer patients.^36^ In HL patients, borderline and major anxiety was found to be more common in women,^40^ but other studies suggest that this association may not be robust.^25^, ^38^, ^40^, ^44^ Although in univariate analyses, women were found to report higher levels/cases of anxiety, gender was not found to be a significant predictor of anxiety in multiple logistic regressions with other sociodemographic and disease characteristics.^25^ Moreover, in the study by Gil‐Fernández and colleagues,^38^ being female was associated with significantly higher likelihood of anxiety caseness compared to males, but difference in mean scores between (1) female and male patients and (2) female patients and female controls were not significant. Similarly, Varela and colleagues^50^ found that a greater number of female HL survivors met the full criteria for PTSD compared to female sibling controls, but this difference was only approaching significance (p = 0.057). In contrast, partial‐PTSD seemed more prevalent in both male and female HL survivors compared to controls, indicating that while diagnosable PTSD may be more common in female HL survivors, partial PTSD may not be limited to one gender.^50^ These studies may suggest a tendency for women with HL to experience anxiety, although it should be noted that one retrospective study found that the association between NHL and anxiety disorders only remained significant in men.^49^ In the same study, HL was found to be associated with depression in both men and women.

#### 3.1.3 Employment Status

Four of six studies suggest that employment status may be significant. One study found that inactive (non‐working) survivors had significantly worse anxiety, depression, perceived stress, perfectionism, general psychiatric wellbeing and sense of coherence compared to active (working) survivors.^28^ Moreover, another study found that lymphoma survivors who undertook professional activity prior to diagnosis were four times more likely to experience clinical levels of fear of cancer recurrence post‐treatment compared to survivors who were not previously engaged in professional activity, even after controlling for gender, age at diagnosis, cancer type, time since complete remission and pre‐existing anxiety/depression.^43^ Indeed, survivors highlighted that being able to return to work was highly valued, especially for patients who were actively working prior to diagnosis.^33^ To some participants, returning to work seem to provide an indication of returning to normality and may also be a source of emotional support/coping.^33^, ^51^ However, two studies suggest that employment status was not found to be associated with mental health status^32^ or anxiety symptoms.^44^

#### 3.1.4 Finance‐related

Three of four studies suggest that finance‐related issues may not be a significant factor. Mental health was not related to socioeconomic status^48^ or income.^44^ One study did find that greater insurance and employment issues was linked to worse PTSD symptoms,^31^ although another study found no relationship between these issues and mental health status.^32^

#### 3.1.5 Education Level

Four of eight studies suggest that education level may be significant. Prospective studies highlight that lower education at study baseline may be a risk factor for anxiety and depression^34^ and use of psychotropic drugs.^41^ There are mixed findings among large cross‐sectional studies. Some studies suggest that lower education may be a significant risk factor of PTSD^31^ and anxiety^25^ whereas other studies found no significant associations with mental health status,^32^, ^48^ distress and anxiety and/or depression.^42^, ^44^

#### 3.1.6 Race/Ethnicity

One of three studies suggest race may be a significant predictor. One study found that non‐whites (69.1% of whom were African‐Americans) were more likely to report PTSD symptoms^31^ whereas other studies found that race was not a significant predictor of anxiety symptoms^44^ or mental health status.^32^ Only one study examined ethnicity (Hispanic and non‐Hispanic) as a predictor, finding no significant relationship between this factor and PTSD symptoms.^31^

#### 3.1.7 Marital Status

Four of six studies indicate that marital status may not be a significant factor of commonly measured forms of distress. Marital status is not a significant predictor of mental health status,^32^ distress,^42^ anxiety and/or depression.^42^, ^44^ One study found that being married/having a partner seemed to buffer against existential concerns such as loss of identity and associating reduced meaning to the disease.^36^ Another study found that in univariate analyses, those who were separated/divorced reported greater anxiety/depression but in multiple logistic regression with other sociodemographic and disease characteristics, marital status was no longer a significant predictor of anxiety or depression.^25^

#### 3.1.8 Having Children

Two of three studies suggest that having children was not significantly associated with anxiety and/or depression symptoms.^25^, ^44^ However, a qualitative study by Monterosso and colleagues^52^ highlighted some participants found that having children around was helpful in coping with the difficulty of resuming to pre‐diagnosis activities.

#### 3.1.9 Habitual Status

One population‐based cross‐sectional study found no significant association between anxiety/depression and whether survivors were living alone, living with parents, or cohabiting at time of diagnosis.^25^

### 3.2 Disease Characteristics

Seven disease‐related characteristics were examined: presenting symptoms (n = 11), symptom burden (n = 8), histological diagnosis (n = 7), disease stage (n = 4), type of treatment (n = 12), survivorship period (n = 9) and time since diagnosis/treatment (n = 7).

#### 3.2.1 Presenting Symptoms

Eight of eleven studies suggest that presenting symptoms may influence distress. Four out of five studies suggest that comorbidity may be a significant factor risk factor for anxiety and depression over time,^34^ the use of psychotropic drugs over time,^41^ worse mental health status^31^ and greater PTSD symptoms.^32^ However, one study found that patients with clinical levels of fear of cancer recurrence did not present mental/behavioural disorders more frequently nor have a higher Charlson Comorbidity Index compared to patients without clinical levels of fear of cancer recurrence.^43^

Only one out of three studies found B‐symptoms to be a significantly robust factor. Presenting with B‐symptoms was found to be consistently associated with distress, anxiety and depression, regardless of whether DT or Hospital Anxiety and Depression Scale (HADS) was examined continuously or categorically.^42^ In the same study, B‐symptoms remained the only significant predictor of clinically significant distress in a multiple logistic regression model with ‘disease stage in patients' opinion’ and ‘interval from time of diagnosis’.^42^ However, in a study examining predictors of distress among HL survivors, univariate analyses indicated that B‐symptoms may be a risk factor of depression but this association was not robust—in multiple logistic regression with other sociodemographic and disease characteristics, B‐symptoms was no longer a significant predictor of anxiety or depression cases.^25^ Similarly, patients with B‐symptoms did not report greater depression compared to controls, and although they tended to report greater anxiety, this difference was not significant.^38^

All three studies suggest that fatigue may be a significant issue for survivors and patients undergoing active treatment. Patients described feeling powerless, isolated, and trapped in their own bodies and home as they felt fatigued from chemotherapy treatment.^53^ Among patients undergoing autologous stem cell transplant, all domains of fatigue were found to be strongly correlated with depression.^54^ Survivors also highlighted that fatigue significantly impacted their capacity to socialise, work or spend time with family.^33^

#### 3.2.2 Symptom Burden

All eight studies highlighted how the experience of symptoms may contribute to distress. During pre‐diagnosis, whether symptoms were acute or vague seemed to influence the type of emotional distress experienced—for patients who experienced sudden/acute symptoms, anxiety, fear and panic were prevalent; for patients who experienced long‐term/vague symptoms, a feeling of general unease better described their emotions.^55^ A prospective study by Øvlisen and colleagues^41^ found that an ECOG performance score of >1 and a higher International Prognostic Index (IPI) predicted greater risk for psychotropic prescriptions, which highlights that poor physical functioning and prognosis may contribute to distress. Indeed, a large cross‐sectional study found that among the domains outlined in NCCN's Problem List, physical symptoms were one of the significant predictors of clinically significant distress in Chinese patients.^42^ Night sweats and itching have been reported by participants as highly interrelated with other physical/psychological symptoms.^56^ Another study showed that people who experienced symptoms of both distress and nausea during treatment were more likely to report the experience of distress or nausea in reaction to cues reminiscent of cancer treatment up to 20 years post‐treatment.^57^ However, one study found that despite the increased symptom distress during active treatment (i.e. side effects of treatment adding on to the disease symptoms), patients perceived an improvement in overall health.^56^

Some studies also reported the social impact of physical symptoms.^29^, ^56^, ^58^, ^59^ Johansson and colleagues^56^ found that itching was particularly distressing as it made patients constantly irritable and some participants commented that to be seen scratching may feel dehumanising. One study found that hair loss made many participants in early aftercare feel ashamed and constantly reminded about their disease, contributing to an increased emotional burden.^59^ Ruan and colleagues^58^ examined how lymphoma impacted the everyday lives of patients, finding that appearance‐altering symptoms generated negative self‐image and was reported to negatively impact patients' interpersonal relationships and career development. Similarly, female survivors who had difficulty accepting their altered appearance reported poorer self‐confidence over time.^29^ Apart from appearance changes, Matheson and colleagues^29^ also found that many male survivors struggled with having to temporarily depend on others for support because this contrasted with their usual role of providing, instead of receiving, support.

#### 3.2.3 Histological Diagnosis

Four of seven studies found no significant association between type of lymphoma and distress. It was not a significant predictor of anxiety and/or depression,^42^, ^44^ fear of cancer recurrence^43^ or mental HRQOL.^39^ However, recent longitudinal studies highlight that histological diagnosis may have differential influence on distress. One retrospective study found that both HL and NHL were associated with greater incidence of depression diagnosis over 10 years compared to controls, even when age and gender were accounted for, whereas only NHL was associated with greater incidence of anxiety disorders.^49^ Øvlisen and colleagues published studies examining cumulative incidence of psychotropic drug prescriptions over time among survivors of HL^35^ and NHL.^41^ Across these studies, both HL and NHL survivors had increased risk of psychotropic drug use compared to controls. However, the distress trajectory differed among the NHL subtypes—it was found that among aggressive NHL patients, cumulative incidence of such prescriptions increased sharply during the first 6 months after diagnosis but gradually normalised over time whereas increase in psychotropic drug use for patients with indolent and intermediate NHLs was gradual and extended such that risk of medication use remained higher for indolent NHL patients even after 5 years.^41^

#### 3.2.4 Stage of Disease

Three of four studies did not find disease stage to be a significant predictor of distress.^25^, ^26^, ^38^ One study did find that disease stage *in patients' opinions* may be associated with distress, anxiety and depression, but this association was not robust and became non‐significant when included in the same model with B‐symptoms.^42^

#### 3.2.5 Type of Treatment

Five of ten studies found that type of treatment may be a significant factor. Receiving a transplant,^32^, ^36^ receiving chemotherapy^60^ or having higher radiation therapy doses^40^ were linked to greater distress. One study also found that patients who underwent radiotherapy were more likely to report better social functioning and mental health, but the authors suggested that no conclusion should be drawn from this observation as patients had localised disease at diagnosis.^48^ However, other included studies generally did not find a significant relationship between type of treatment and distress.^25^, ^26^, ^38^, ^42^, ^44^

#### 3.2.6 Survivorship Period

Eight of nine studies suggest survivorship period to be a significant predictor of distress,^26^, ^29^, ^33^, ^41^, ^44^, ^52^, ^60^, ^61^ with only one study finding no significant relationship.^25^

Being on active treatment was associated with greater actionable distress^26^ whereby having relapsed may predict significantly worse activity impairment, mental wellbeing, anxiety and depression symptoms^60^ and greater use of psychotropic drugs.^41^ Similarly, for survivors, having a history of relapse was associated with greater anxiety symptoms.^44^ Survivors with follicular lymphoma who were in remission but not considered disease‐free also reported greater anxiety symptoms but not depression symptoms.^60^ Even so, one study did not find any difference in anxiety/depression levels between those who relapsed and those who did not relapse.^25^ However, it should be noted that majority of participants in the relapsed group did not have active disease when the study was conducted (n = 29), with five participants having relapsed with current disease.

Qualitative studies have also highlighted the difficulty of transitioning into survivorship immediately post‐treatment. This period has been characterised by feelings of isolation and uncertainty^33^, ^61^ as participants experience an abrupt end to professional care/monitoring,^29^ losing a sense of security; and being left wondering about next steps.^52^ Indeed, Hackett and colleagues found that many participants reported feeling ‘unprepared and alone’ whereby those who provided more positive descriptions were on maintenance treatments or had more frequent visits due to transplant follow‐up.^33^

#### 3.2.7 Time Since Diagnosis/Treatment

Seven of nine studies suggest that distress varies over time, but the pattern of specific distress trajectories remains unclear. Later time since complete remission was associated with greater likelihood of experiencing clinical levels of fear of cancer recurrence, even after individual characteristics were controlled for.^43^ In a study with survivors averaging 7 years post‐diagnosis, longer time since diagnosis was associated with less cancer worry but more grief and guilt over the deaths of other survivors.^36^ A cross‐sectional study found that depression and anxiety seemed to peak between 7 and 10 years post‐diagnosis while survivors 3–6 years post‐diagnosis reported the lowest distress, and anxiety and depression seem to reduce over time between 11 and 23 years post‐diagnosis.^25^ This was further tested in multiple logistic regression with other sociodemographic and disease characteristics, whereby ≥7 years post‐diagnosis remained a significant predictor of anxiety caseness but not depression caseness. Among young adult survivors with HL, incidence of severe depression seems to improve over time such that there were no reports of severe depression at 6 months, whereas prevalence of severe anxiety reduced at 1 month post‐treatment but appear to gradually increase at 3 months and 6 months.^27^ Longitudinal studies comparing the cumulative incidence of psychotropic prescriptions between lymphoma patients and matched controls found that for both HL and NHL patients, the risk of psychotropic drug use generally normalises over time such that at 5 years post‐diagnosis, survivors had similar rates of psychotropic prescription as the controls.^35^, ^41^ On the contrary, two studies with survivors ≥2 years post‐diagnosis/treatment did not significantly predict mental health status^32^ or anxiety symptoms.^44^

### 3.3 Psychosocial Factors

Six psychosocial factors were examined: previous experience of mental ill health (n = 3), cognitive functioning (n = 2), lifestyle factors (n = 2), social support (n = 11), adjustment to cancer (n = 7) and life changes/experiences (n = 4).

#### 3.3.1 Previous Experience of Mental Ill Health

Three studies suggest that having previously had mental ill health is a significant factor. Survivors who reported greater anxiety and depression scores at baseline were more likely to experience clinical levels^†^ of fear of cancer recurrence, although it should be noted that comorbidities such as mental and behavioural disorders did not differentiate between clinical and non‐clinical levels of fear of cancer recurrence.^43^ Further, having psychiatric symptoms prior to treatment was a robust predictor of anxiety and depression after treatment, even when sociodemographic and disease characteristics were accounted for.^25^ Some studies suggest that being emotionally distressed during treatment may also contribute to distress after treatment. Having psychiatric symptoms during treatment predicted anxiety caseness,^25^ and survivors who experienced symptoms of both distress and nausea during treatment were more likely to experience distress and nausea when exposed to treatment‐related cues.^57^

#### 3.3.2 Cognitive Functioning

Two studies examined cognitive functioning and suggested a significant relationship. Survivors with impaired cognitive functioning reported greater symptoms of distress, anxiety, stress, poor sleep quality and lower QOL compared to survivors without impaired cognitive functioning.^63^ Additionally, some survivors in early aftercare reported that having cognitive problems made them feel like a burden to their partners.^59^

#### 3.3.3 Lifestyle Factors

Two studies found significant results. One study found that meeting health recommendations was associated with greater mental HRQOL and lower post‐traumatic stress.^64^ Moreover, survivors who had poor sleep quality had impaired HRQOL and reported significantly worse HRQOL compared to participants with good sleep quality.^65^

#### 3.3.4 Social Support

All eleven studies examining social support suggest it may be a significant mitigator of distress. Less social support is linked to greater PTSD symptoms^31^ and has been found to consistently predict worse mental health status, QOL and fatigue, above and beyond impact of cancer and/or sociodemographic/disease characteristics.^32^, ^48^ Similarly, a larger social network seems generally associated with improved scores on domains such as bodily pain, social functioning, mental health, emotional role and overall cancer‐specific QOL.^48^ Among qualitative studies that highlighted social support, participants have reported it was helpful and highly valued.^29^, ^33^, ^51^, ^52^, ^59^, ^61^, ^66^, ^67^ Specifically, social support was helpful in mitigating fear and anxiety,^66^ and family members and friends often helped to reinforce patients' own resources and validated patients' experiences.^51^ Further, relationships were reported to strengthen after cancer diagnosis,^33^ and some participants also felt that meeting co‐patients enabled them to expand their circle of friends.^51^

However, these qualitative studies also provided further nuance. Some participants have reported feeling like a burden to their support networks.^66^ Although family and friends were generally supportive, some relationships became tense or were lost as loved ones were unsure about how to react to the diagnosis,^33^ leaving patients feeling alone and disappointed.^51^ Moreover, family and friends could be dismissive of the patients' situation/experience,^52^ with participants commenting that this may be partly due to the lack of outward signs of disease.^61^ To this end, peer support groups or companionship with other patients were highly valued,^29^, ^33^, ^52^, ^61^ but not always easily accessible post‐treatment^33^, ^61^ and some participants would rather not participate as they preferred to move away from the cancer label.^52^ Some participants also commented that peer support groups may not be helpful as seeing peers becoming unwell makes it difficult to remain positive about their own prognosis.^61^

#### 3.3.5 Adjustment to Cancer

Seven studies suggest that the way patients/survivors make sense of their cancer experience may influence distress. Negative appraisals about how life‐threatening and intense cancer and its treatment was, predicted for worse PTSD symptoms.^31^ Similarly, negative appraisals predicted mental health status above and beyond other sociodemographic and disease characteristics, although this association became non‐significant when positive/negative impact of cancer was accounted for.^32^ Misperceptions may also influence how survivors engage with care services and make sense of life post‐treatment. In a focus group study consisting of NHL survivors, participants shared the perception that haematological cancer was less like a cancer diagnosis but more akin to a chronic illness.^61^ This resulted in these survivors feeling that cancer supportive care services were not relevant to them and feeling isolated from peers with other cancers and their family and friends.

On the contrary, positively reframing the cancer experience, practicing acceptance and mental disengagement may be helpful cognitive strategies that can facilitate acceptance.^29^, ^51^, ^67^ For example, during treatment, some patients focused on finding ‘opportunities in difficulties’ and distracted themselves from the disease and its treatment by engaging in activities such as work and physical activity.^51^ Young adults often engaged in positive reframing by holding on to positive perceptions of HL and its trajectory, making social comparisons to others who may have ‘worse cancers’ and finding reasons to suggest that the timing of cancer at young adulthood can be advantageous.^29^ However, some participants shared that seeing others struggle was difficult and guilt may be evoked when others were perceived to be coping with worse situations.^61^

Other lymphoma patients/survivors may find it more helpful to take control of the situation. A cross‐sectional study examining the relationship between use of complementary and alternative medicine (CAM) and HRQOL found that CAM users have a significantly greater mental health status than non‐users, and this relationship was fully mediated by perceived personal control.^30^ Similarly, some patients found it helpful to take control by managing cancer treatment like a project,^51^ and some survivors preferred to plan ahead (e.g. figuring out first steps if cancer does recur).^67^ However, surrendering control to clinicians was helpful for others.^61^

Apart from the abovementioned cognitive strategies**,** lymphoma survivors also described religious/spiritual coping and restraint coping (i.e. holding themselves back from prematurely worrying) as helpful in keeping fear of cancer recurrence under control.^67^

#### 3.3.6 Life Changes/Experiences

Two studies primarily examined how life changes influence distress. Negative life changes seem to be a strong predictor of lower mental HRQOL whereas findings are mixed on whether positive life changes significantly predict mental HRQOL.^32^, ^37^ Other studies highlight that life experiences may be an overarching factor influencing other factors of distress. For example, a qualitative study found that patients' life experiences and how they dealt with these life experiences may shape their coping strategies during and after cancer treatment.^51^ The study also found that while many patients struggle with after‐treatment late effects and fear of relapse, patients who have experienced difficult situations earlier in life and/or retirees seemed to find it easier to manage post‐treatment uncertainty.^51^ This is further supported by the finding that in a model predicting mental health status, the relationship between type of treatment and negative appraisals of life threats became non‐significant after the addition of the Impact of Cancer scale.^32^

### 3.4 Healthcare Factors

Two factors were examined, with majority of findings informing the role of healthcare professionals in mitigating distress (n = 11) and four studies specifically examining routine events within healthcare.

#### 3.4.1 Healthcare Professionals

Eleven studies stressed the importance of patient–clinician relationship and communication from pre‐diagnosis up to posttreatment and survivorship.^29^, ^33^, ^44^, ^52^, ^53^, ^55^, ^59^, ^61^, ^66^, ^67^, ^68^

Better patient–physician relationship predicted lower mean anxiety, above and beyond history of relapse.^44^ Participants greatly valued clear communication of individually tailored information^61^ and the sense of security from being monitored closely by a team who genuinely cares for them.^52^ Indeed, a prospective study found that for survivors ≥2 years post‐diagnosis, satisfaction with information provided at baseline was important for their physical and emotional functioning.^68^ Moreover, participants who were more stably satisfied across time reported better emotional and cognitive functioning and lower levels of anxiety and depression.^68^ A study found that one of the two main sources of fear of cancer recurrence was aspects associated with relapse or secondary cancers, which included cognition/feelings such as ‘a general feeling that there could be undetected cancer cells’.^67^ This may highlight the importance of reassurance from healthcare professionals. Indeed, in the same study, participants reported that oncologists' statements that were most helpful in reducing fear of cancer recurrence include statements about low recurrence rates, recommendations of specific action plans, communication about treatment progress, reassurance of continuity of care and oncologists' general positive attitude, honesty, and expertise.^67^

However, participants often commented that their emotional/psychosocial needs are not addressed.^52^ Factors such as clinicians' unsympathetic personalities and feeling uninformed have made participants feel disconnected from the clinical care team^66^ and busyness of the clinical environment made it difficult for survivors to express their unmet needs.^61^, ^66^ These issues are commonly highlighted during the transition into survivorship. To this end, a qualitative study examined participants experiences of aftercare consultation whereby a clinician would contact participants shortly after treatment and support them in transitioning into aftercare.^59^ It was found that empathy and understanding participants and their life stories made participants feel better supported in this period.^59^

#### 3.4.2 Healthcare Events

Three of four studies suggest that routine healthcare events such as follow‐up appointments and decision‐making may be a source of distress. Participants reported that fear of cancer recurrence intensifies leading up to follow‐up visits^33^ and in the period between undergoing a surveillance scan and receiving results.^44^ Hackett and Dowling^33^ also found that fear of cancer recurrence was less intense in individuals who had indolent lymphoma, some of whom have experienced a recurrence, and it was generally more intense in the first two years post‐treatment but reduced with time. Another study examining whether treatment decisions may contribute to distress found that follicular lymphoma survivors reported mild depression, moderate anxiety and moderate‐to‐severe distress, but seemed largely satisfied and unconflicted about their recent treatment decisions.^69^

## 4 DISCUSSION

Past reviews about distress in lymphoma patients have focused on prevalence of distress or HRQOL more generally, making it difficult to delineate the factors of emotional distress specific to this population. Identifying these factors is crucial for improving the current approach of distress screening, equipping clinicians with information about who for, where and when might psychosocial services be most necessary.

### 4.1 Consistent Factors

#### 4.1.1 Who?

Across 41 manuscripts included in this review, consistent risk factors of distress include younger age, relapsed disease, and greater comorbidities and symptom burden. Adequate social support, adaptive adjustment to cancer, engaging in work and healthcare professionals' support may be important in mitigating distress. These findings align broadly with reviews on lymphoma and other cancers. Younger age has consistently been associated with distress across cancers,^70^ including haematological cancers.^71^, ^72^ Physical impairment was consistently linked to anxiety and depression in ovarian cancer patients,^73^ and some evidence suggest that poor physical health pre‐diagnosis or postoperative can be linked to long term distress in breast cancer patients.^74^ Lymphoma survivors with a history of recurrent lymphoma are suggested to be especially at risk of anxiety.^13^ Cancer guidelines also highlight that lack of social support is a risk factor for distress across various cancers.^7^ A meta‐ethnography of lymphoma survivors' experiences highlighted the importance of social support, including the support from healthcare providers, throughout acute and chronic survivorship.^75^ Furthermore, there is some evidence that adaptive coping strategies and positive perceptions/appraisals of disease and physical/mental functioning are associated with better QOL in haematological cancer patients.^76^ Going forward, future research could focus on examining the abovementioned factors in combination. A combination of these factors may point to marginalised populations such as older adults and minority groups (e.g. culturally and linguistically diverse (CALD) population) as requiring additional support. For example, studies suggest that CALD cancer patients/survivors often experience worse health outcomes,^77^ and this may be attributed to inequities inherent in the health system (e.g. cultural insensitivities and language barriers) which make access and navigation challenging for this population.^78^

Adding to current literature, this review highlights that although younger age is often linked with distress, older age may be linked to greater depression. Although indirectly, this does align with some studies highlighting that among cancer patients, older age may buffer against anxiety but not depression.^79^ This could be explained by emerging research highlighting the differential impact of cancer across the lifespan of patients. AYAs and midlife survivors are often more concerned about issues relating to school, finances, employment and sexuality/intimacy compared to adults 65 years and above.^72^, ^80^ Contrastingly, older adults commonly report concerns about physical functioning/symptom distress and loneliness,^81^, ^82^ with one study indicating that reduced social support, increased comorbidity and advanced disease stage is associated with high levels of depression in older adults across cancer sites.^79^ Considering that distressed older adults are often less likely to use mental health services compared to distressed younger adults,^71^ these findings demonstrate the need for more studies examining distress specifically in older age groups. In this review, only three studies had majority of participants aged ≥65,^30^, ^31^, ^32^ and one qualitative study engaged in purposive sampling to ensure representativeness of each age group.^33^

Additionally, this review highlighted that lymphoma patients/survivors made sense of and coped with the lymphoma experience in various ways. Negative appraisals of cancer and its treatment were linked to worse PTSD symptoms and mental health status. This aligns with literature in haematological cancers more broadly.^76^ The studies in this review also suggest that coping strategies such as positive reframing, taking/relinquishing control, mental disengagement, religious/spiritual coping and restraint coping may be helpful. However, more quantitative studies should be done to clarify how these coping strategies link to distress. For example, there is some evidence that haematological cancer patients with higher scores in Helplessness‐Hopelessness and lower scores in Fighting Spirit may be at risk of greater distress and worse QOL,^76^ above and beyond age, type of treatment, and satisfaction with information provided.^83^

It may be pertinent to further explore how mental disengagement and work relates to distress. From this review, it appears that among professionally active lymphoma survivors, there is a desire to return to work and returning to work may be beneficial for survivors' emotional wellbeing, although fear of cancer recurrence could be experienced. However, there is some evidence that across cancer survivors, physical and psychosocial problems (e.g. physical functioning/appearance, cognitive limitations, fatigue and coping issues) appear to persist beyond the return‐to‐work period.^84^ As this review only included one significant cross‐sectional study with long‐term survivors,^28^ and another small mixed‐methods study which did not find significant effects,^44^ there is a need for prospective studies to examine the emotional wellbeing of working lymphoma survivors over time. Moreover, one study which did not find a significant relationship between employment status and mental health status was a large cross‐sectional study among NHL survivors whereby the average age of participants was 62.9 years and 46% of participants were greater than 65 years old.^32^ This supports the movement towards supporting survivors in achieving individually tailored ‘work‐related goals’ instead of a general ‘return to work’.^85^ Future research could explore if there are common ways in which non‐working survivors and/or older adults return to normality or mentally disengage.

#### 4.1.2 Where/When?

Studies from this review found that being in active treatment was associated with clinically significant distress,^26^ as this may be a period of significant symptom burden with patients experiencing both the symptoms of disease and side effects from treatment^56^; and some evidence that survivors may experience distress and nausea when exposed to treatment‐related cues up to 20 years posttreatment.^57^ Similarly, a review of psychological outcomes of lymphoma suggest that fear of cancer recurrence experienced by survivors often stemmed from the fear of having to undergo treatment again, suggesting a link between treatment toxicity and distress.^12^ To this end, Vargas‐Román and colleagues have suggested that music interventions may help relieve pain and anxiety in lymphoma patients.^13^

Our review also found that apart from functional impairment, lymphoma patients/survivors may have appearance and social concerns due to the physical changes associated with lymphoma and its treatment. This aligns with a review of distress factors in breast cancer patients^86^ and may suggest that lower self‐esteem associated with appearance changes (e.g. hair loss and weight gain) is not only experienced by females. To this end, body image interventions have been found to be beneficial^87^ and may warrant further research in this population. Apart from appearance concerns, one study found that the temporary dependence on others was challenging for young adult male lymphoma survivors due to their usual role of being the one that others depend on.^29^ This suggests that further studies on exploration of existential concerns around identity and life purpose may be beneficial.

Importantly, several qualitative studies in this review highlight the need for support in the transition into survivorship immediately posttreatment.^29^, ^33^, ^52^, ^61^ This supports the importance of having survivorship care plans in place to guide coordination and conversations around after care.^88^ Among lymphoma survivors, phase I and II trials of nurse‐led survivorship care programs seem promising.^89^, ^90^ Aligning with Vargas‐Román and colleagues'^13^ review highlighting that anxiety peaks around follow‐up appointments, the current review also demonstrated that surveillance scans are a source of fear of cancer recurrence in this population^33^, ^44^, ^67^ and there is a general trend for distress around these scans to reduce over time.^33^ These findings suggest that in these survivorship care plans, it may be beneficial to include information around how to cope with the process and uncertainty around these routine scans/appointments, especially in the first few years after treatment.

Generally, this review also suggests that patients' interaction with their physicians may play a large role in influencing patients' distress. Good patient–physician relationship and satisfaction with information provided were associated with lower anxiety/depression and generally, clear communication of individually tailored information, provision of reassurance, and continuity of care seems appreciated by patients/survivors. However, unmet emotional/psychosocial needs were also reported in this review. To this end, guidelines on how distress can be recognised and managed in patient–physician conversations^91^, ^92^ and through screening tools^15^, ^93^ may be beneficial.

### 4.2 Generally Non‐Significant Factors

Interestingly, gender does not seem to be a robust predictor of overall distress among lymphoma patients/survivors, although there seems to be a tendency for women with HL to experience anxiety. This contrasts with a review in HRQOL of long‐term lymphoma survivors which found that male survivors reported better emotional functioning,^17^ and literature across cancer types suggesting that women/females are more likely to experience distress^94^ and depression.^19^ However, this pattern does align with reviews focusing specifically on HL or NHL: gender was found to be a significant predictor of distress when distress was examined among HL survivors^10^, ^18^ whereas Leak and colleagues^8^ review of QOL among NHL survivors did not highlight gender as a significant predictor. Considering that HL has a generally better prognosis/curability rate than NHL, this may highlight that distress in NHL patients/survivors is not gender‐specific. Future research could examine whether communication of prognosis might affect distress. Moreover, across studies, gender was conceptualised as a binary construct (i.e. men/women). Future studies should explore how other gender identities may relate to distress.

Marital status was generally not found to be a significant predictor of mental health status and distress, although it may buffer against existential concerns. This aligns with other reviews indicating that marital status is not linked to anxiety/depression in HL survivors^10^ nor psychiatric morbidity in cancer patients.^95^ Taken together with nuances in social support found in this review, this may indicate that marital *quality* may be more important to assess than marital status, providing a direction for future research in this factor.

### 4.3 Ambivalent Findings

Mixed findings were observed for type of treatment, histological diagnosis, time since diagnosis/treatment and education level. This does align with the fact that few reviews examining HRQOL or distress in this population have highlighted disease/treatment characteristics as consistent predictors of distress. Although NHL survivors who received chemotherapy were found to report worse mental health functioning/wellbeing,^8^ and having some treatment (e.g. with rituximab) was found to be psychologically beneficial compared to a watch‐and‐wait strategy in lymphoma,^13^ Cook and colleagues' review found that type of treatment was not associated with long‐term distress in cancer survivors.^74^ Regarding histological diagnosis, three recent longitudinal studies from this review highlight that NHL subtypes may have differential influence on distress—unlike HL patients/survivors, NHL patients/survivors may experience greater anxiety compared to controls; and aggressive lymphomas and indolent lymphomas may have different distress trajectories.^35^, ^41^, ^49^ This might explain the inconclusive findings in the link between time since diagnosis and distress. Future studies focusing on larger mixed samples or on specific subgroups may be useful in delineating the distress trajectory across HL/NHL subtypes and clarifying at which timepoint might patients/survivors be most prone to distress. Education level was examined in eight studies whereby the four studies which demonstrated a negative relationship between education level and distress were either longitudinal or had a large sample size. Future studies may benefit from examining clinical significance instead of statistical significance.

Generally psychosocial/environmental factors including having children, habitual status, race/ethnicity, finance‐related factors, previous mental ill health, cognitive functioning, lifestyle, life changes/experiences and routine healthcare events have insufficient studies (n ≤ 4), and therefore may benefit from further research. Particularly, a recent review of prospective studies across cancer sites found that distress and neuroticism at baseline predicted distress 12 months later.^74^ This suggests a need to further explore emotional dispositions and personality in this population. Moreover, in the same review, the authors found limited evidence that typical sociodemographic, clinical or psychological factors could consistently predict long‐term distress.^74^ In the present review, only three studies have examined predictors from baseline,^35^, ^43^, ^68^ highlighting the need for more research in this space. Studies from the present review also suggest life changes/experiences may shape how people appraise and cope with cancer [e.g. 49, 50], underscoring the importance of understanding patients holistically. In addition, considering the significance of survivorship period highlighted from this review, more studies are needed, focusing on other stages of cancer survivorship apart from active treatment and survivorship. In this review, only two studies explored the pre‐diagnosis phase.^55^, ^56^ Furthermore, there appears to be a dearth of research in factors of distress in the end‐of‐life stage.

**Figure 2:**
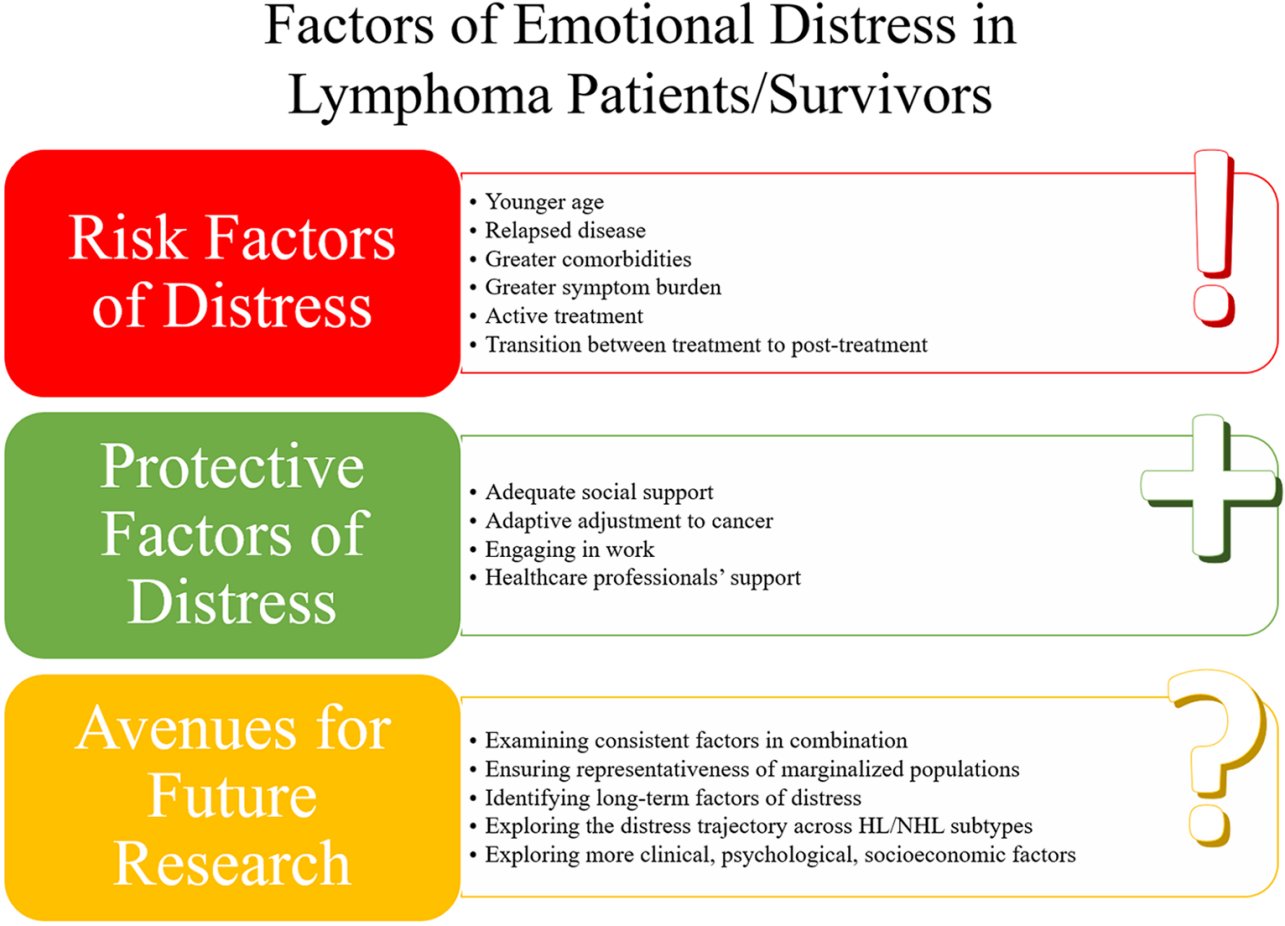
Summary of Key Findings.

Figure 2 below outlines the key findings from this review:

### 4.4 Clinical Implications

The factors identified in this review might support healthcare professionals in identifying at‐risk populations and points of distress where additional support may be beneficial. For example, marginalised groups who often present with a combination of these factors might benefit from more support. Going forward, future research should examine these factors combined and ensure greater representativeness of these populations. Moreover, the identified points of distress also highlight a need to address symptom burden, develop survivorship care plans and improve communication with patients. The review revealed knowledge gaps, especially regarding clinical, psychological and socioeconomic factors of distress, long‐term factors of distress, and the distress trajectory across HL/NHL subtypes. Lastly, this review highlights a need for data on distress and its factors to be collected over time in registries. This might support clinicians in identifying individuals at‐risk of distress while providing longitudinal data for future research.

### 4.5 Limitations

There was heterogeneity in the measures used to examine both distress and its factors; thus, caution should be taken when interpreting the results of this review. Moreover, majority of the studies were conducted in the United Kingdom or the United States, limiting the generalisability of this review to individuals in other countries with different cultures and healthcare practices. Similarly, majority of reviewed studies may only be representative of individuals who are fit to participate in the research process of these studies. It should be also noted that some studies derived their sample from survivorship care programs^43^, ^59^ or clinical trials.^40^, ^67^ Participants in these studies may have received greater support and monitoring compared to other participants.

## 5 CONCLUSIONS

This review is the first to comprehensively synthesise factors associated with distress in lymphoma patients/survivors. Individuals who are younger, have greater comorbidities, greater symptom burden or have relapsed may be at greater risk of distress. Generally, distress may be particularly high during active treatment and in the transition into survivorship, after which distress may only spike around follow‐up appointments. Adequate social support, adaptive adjustment to cancer, engaging in work and healthcare professionals' support are valued buffers of distress. These identified factors may support clinicians in identifying distressed patients/survivors and providing interventions where necessary. A need to routinely collect data on distress and its factors in registries is highlighted. While several consistent factors of distress align with that of other cancers, more research is needed to identify significant factors of distress in lymphoma patients/survivors. Future research should focus on examining the consistent factors in combination, ensuring representativeness of marginalised populations, identifying long‐term factors of distress, exploring the distress trajectory across HL/NHL subtypes and exploring more clinical, psychological and socioeconomic factors.

## AUTHOR CONTRIBUTIONS

**Kai Ping Tan:** Conceptualization (supporting); data curation (lead); formal analysis (lead); investigation (lead); methodology (equal); project administration (lead); writing – original draft (lead). **Dipti Talaulikar:** Conceptualization (equal); funding acquisition (lead); methodology (equal); project administration (supporting); supervision (equal); validation (equal); writing – review and editing (lead). **Brett Scholz:** Conceptualization (equal); methodology (equal); project administration (supporting); supervision (equal); validation (equal); writing – review and editing (supporting).

## CONFLICT OF INTEREST STATEMENT

The authors have no relevant financial or non‐financial interests to disclose.

## ETHICS STATEMENT

Not applicable.

## Supporting information


**Data S1:** Supporting information

## Data Availability

The authors confirm that the datasets supporting the findings of this review are available within the article and/or supplementary materials. Further information on data related to the findings of this study is available upon request from the corresponding author (DT).

## Endnotes

*
*Caseness* defined as a high probability of being diagnosed with psychiatric disorder (e.g., anxiety or depression) based on cut‐off score on standardised scale^46^

†
*Clinical level* of fear of cancer recurrence has not been explicitly defined in literature. This review follows the definition in Simard and Savard, which was guided by DSM‐IV's definition for mental disorders: “a clinically significant behavioural or psychological syndrome, associated with significant distress or disability”^62^, p. 483
