# Supplementary material for: Factors of emotional distress in lymphoma: A systematic review
Source: Cancer Med. 2023 May 17;12(13):14646–62. doi: 10.1002/cam4.6069 (PMC10358206; doi:10.1002/cam4.6069)
Supplement: Supplementary file 1 — Data S1: Supporting information [file CAM4-12-14646-s001.docx]

# Supplementary Material 1

# Factors of Emotional Distress in Lymphoma: A Systematic Review

Kai Ping Tan^1^, A/Prof Dipti Talaulikar^1^, Dr Brett Scholz^1^

^1^ School of Medicine and Psychology, College of Health and Medicine, Australian National University, Canberra, Australia.

Corresponding Author: A/Prof Dipti Talaulikar

Email address: [dipti.talaulikar@anu.edu.au](mailto:dipti.talaulikar@anu.edu.au)

**Table 1**

*Summary of Quantitative Studies*

|  | **Author(s), year (Country)** | **Aim** | **Design; Relevant Statistical Analysis** | **Sample characteristics** | **Factors Measured** | **Distress Outcome Measured** | **Relevant Findings** |
| --- | --- | --- | --- | --- | --- | --- | --- |
| 1. | Bellizzi et al., 2007 (USA) [1] | To examine the positive and negative life changes after cancer, factors related to positive and negative life changes, and how these changes are associated to mental and physical functioning | Cross-sectional; Multivariable linear regression | 308 aggressive NHL survivors. 2 to 5 years post-diagnosis; mean 3.5 years, SE = .1 Mean age = 60 years, 23-85 years. Majority had chemotherapy as part of their treatment (94.2%) and self-identified as non-Hispanic white (inferred, frequency not reported). | Life Impact Scale  Life Orientation Test-Revised  MOS-SSS | SF-36 | Negative life changes consistently predicted lower physical and mental HRQOL when adjusted for sociodemographic, disease, and psychosocial characteristics. Positive life changes did not remain a significant predictor after addition of negative life changes in model.  Socio-demographics correlates of positive and negative life changes:   - Being older predicted lower positive and negative life changes - Higher social support associated with greater positive life changes overall, and lower negative life changes in relationship and financial domains - Optimism associated with lower negative life change but not greater positive life change - Higher education predicted greater positive change in health behaviours |
| 2. | Cameron et al, 2007 (USA) [2] | To examine the prevalence and correlates of persistent aversive symptoms within the classical conditioning framework among HL survivors | Cross-sectional correlational; Logistic regression | 273 HL survivors. 5 years post-treatment (median). Participants were treated with CALGB trials between 1966 and 1986, were part of larger QOL study (Kornblith et al., 1990, 1992), and have no active disease. Median age = 37 years, range = 20-66 years. Mean age at initial treatment = 29 years, SD = 9.9, range = 5-56. Most participants were White (95%), diagnosed with Stage III or IV disease (88%), and treated with chemotherapy alone (80%). | Semi-structured interview prompting participants to report:   - severity of symptoms (i.e., feelings of distress, nausea, vomiting that occurred in past 6 months in reaction to smells, sights, or tastes that reminded them of their cancer treatment) - whether treatment made participants sick during treatment - whether they experienced the symptoms before getting therapy   7-items Psychological Distress subscale in Psychological Adjustment to Illness Scale-Self Report (PAIS-SR)  Ratings of emetic potential across 39 treatment regimens by five oncologist-haematologists (cluster analysis used to group regimens into high, moderate, or low emetic potential) | | Factors of persistent symptoms**:**   - Persistent symptoms more common in people who reported developing both anticipatory symptoms of distress and nausea during treatment (OR = 2.65 – 8.70; p <.05 - <.01), and people who were generally more psychologically distressed (OR = 1.08 – 1.27; p <.05 – p <.01). - Persistent symptoms of distress more often triggered by sights reminiscent of treatment, whereas persistent nausea more often triggered by smells. These cues may not be specific to treatment. They can be general cues such as route to hospital, smell of perfume or cleaning agents. - Being treated more recently was associated with conditioned distress cued by sight/smell (OR = .20 – 22; p < .05 – p <.01). Being treated more recently was also related to conditioned nausea cued by sights (≥5 years vs. < 2 yrs, OR = .19, p < .01), but not nausea cued by smells. - Emetic potential of treatment (i.e., whether the treatment will cause ppl to vomit) was not a significant predictor of conditioned distress or nausea. |
| 3. | El-Banna et al., 2004 (USA) [3] | To describe patterns of and relationship between depression and fatigue in lymphoma patients undergoing autologous peripheral blood stem cell transplantation (PBSCT) | Prospective correlational; Friedman's repeated measures analysis of variance on ranks, Spearman’s correlation | 27 lymphoma patients undergoing PBSCT. Mean age = 49 years, SD = 13.71, range = 19-71 years. 89% had NHL whereas 11% had HL. 89% were treated with a chemotherapy protocol consisting of carmustine, etoposide, cytosine, arabinoside, and melphalan (i.e., BEAM) and 11% were treated with BEAM with rituximab. All participants were Caucasian. | Revised Piper Fatigue Scale (PFS)  Centre for Epidemiologic Studies – Depression (CES-D) Scale | | Fatigue and depression increased sharply and peaked 7 days after chemotherapy, then decreased from 7 days to 14 days after chemotherapy. Fatigue scores had a significant increase in all dimensions except for cognitive dimension. Depression scores met cut-off score (>16 points) indicative of major depression from 2 days after chemotherapy.  **Scores of total fatigue and all four dimensions were highly correlated with depression** (rho = 0.842 to .0929, p < .01). Highest reported correlation was between affective fatigue and depression (r = 0.929, p = .001). |
| 4. | Geffen et al., 2003 (Israel) [4] | To evaluate the frequency of full PTSD and PTSD associated symptoms, and to explore the relationship between PTSD and HRQoL | Cross-sectional; Chi-square analysis, ANOVA, MANOVA, Pearson correlation, MANCOVA | 44 long-term lymphoma survivors (≥ 2 years post-treatment and disease-free). Median age 51 years, range = 27-80 years. 81.82% were diagnosed with NHL (61% with large cell/diffuse mixed lymphoma; 14% with grade I/II follicular lymphoma; 5% with small lymphocytic lymphoma; 2% with cutaneous T-cell lymphoma) and 18% with HL. Majority had stage I (34%) or stage II (39%) cancer. 54% of patients underwent chemotherapy, 32% underwent radiotherapy and chemotherapy, and 14% underwent radiotherapy.  44 matched controls who experienced at least one traumatic life event (most common = motor vehicle accidents and sudden death of life partner) recruited from staff of large factory. Median age 50 years, range 23-79 years.  Race/ethnicity not reported. | Demographics  SF-36 (Hebrew validated translation)  Lymphoma survivors compared to matched controls with at least one traumatic life event (i.e., experience of lymphoma compared to other traumatic life events) | PTSD Inventory (Hebrew version) – Partial PTSD defined as presence of 2/3 symptom clusters; Full PTSD defined as meeting diagnostic criteria of all three symptom clusters. | The experience of lymphoma may be equivalent to other traumatic life events, and perhaps could be worse in terms of hyper-arousal symptoms. Moreover, the experience of lymphoma may also predispose individuals to lower physical HRQOL, above and beyond the contribution of PTSD intensity.  Factors for PTSD highlighted:   - Comparison among survivors showed that earlier age of disease onset associated with more intense intrusion (F = 7.7, p < .01) and avoidance symptoms (F = 5.0, p < .05). - Mental functioning not found to be significantly different between survivor and control groups after controlling for PTSD intensity. However, physical functioning was significantly different.   Experience of lymphoma may be as traumatic as other traumatic life events, and perhaps could be worse in terms of hyper-arousal symptoms. |
| 5. | Gil-Fernandez et al., 2003 (Spain) [5] | To examine quality of life and psychological wellbeing in Spanish survivors of HL and compare these to outcomes of healthy controls. | Cross-sectional case-control; Chi-Square, Fisher’s Exact Test, Mann Whitney U-test, ANOVA factorial test | 46 Spanish HL survivors treated between January 1979 to December 1997 without active disease and free of secondary malignancies (median = 7.6 years post-diagnosis). Mean age = 43 years, SD not reported, range = 15-80 years. Mean age at diagnosis = 34 years, SD not reported, range = 13-77 years. 43% had III-IV Ann Arbor stages, 43% with B-symptoms. 63% participants received chemoradiotherapy, 22% received chemotherapy only, 15% received radiotherapy only.  46 matched controls. Mean age = 40.4 years, SD not reported, range = 24-67 years.  Race/ethnicity not reported. | Sociodemographic questionnaire  Experience of lymphoma (HL survivors vs. healthy controls) | EORTC QLQ-C30  HADS – non-cases = <7, doubtful cases = 8-10, caseness (i.e., clinically significant anxiety/depression) = >11 | No differences found between survivors and controls in global state of health and quality of life, but survivors had significantly lower physical function (p = .05) and social operation (p = .0015). Survivors also reported greater dyspnea symptoms (p = .03) and economic difficulties (p = .017) compared to controls. No relationship found between dyspnea symptoms and treatment modality, and all functional/symptomatic scales in EORTC QLQ-C30 were similar regardless of treatment.  No significant difference between survivors and controls in mean scores of proportion of cases of anxiety/depression.  Female survivors and survivors > 45 years old may be at greater risk of anxiety and depression compared to other survivors, but these survivors did not have significantly worse anxiety/depression scores compared to controls**.**  Sociodemographic/clinical risk factors:   - Survivors >45 years old: worse physical operation (p = .02), global appreciation of state of health (p = .04), loss of appetite, diarrhea issues, fatigue (n.s.) and depression (p = .03) compared to survivors <45 years old. When comparing between survivors and controls over 45 years, survivors reported higher depression scores but this was not significant. - Female survivors: Worse physical function (p = .01) and anxiety cases (p = .01). Anxiety scores higher for female survivors than female controls but difference was not significant. - Survivors who presented with B-symptoms at diagnosis had greater tendency towards reporting anxiety caseness, although this is not significant (p = .07)   No significant association found between:   - Dyspnea symptoms and treatment modality - Anxiety and disease stage, type of treatment (including autologous transplant), clinical evolution of disease. - Depression and sex, B symptoms at diagnosis, or disease stage at diagnosis |
| 6. | Hamilton et al., 2012 (USA) [6] | To examine the relationship between use of complementary and alternative medicine (CAM) and HRQOL and test whether perceived control mediated relationship between CAM use and HRQOL | Cross-sectional correlational; Multiple linear regression | 319 aggressive NHL survivors (2-5 years post-diagnosis). CAM-related data and psychosocial variables obtained from population-based study of NHL survivors designed to assess NHL survivors’ use of follow-up care and evaluate their long-term health outcomes [7]. Majority were non-Hispanic white (69.9%), married/living as married (65.1%), and had no evidence of recurrence (80.6%). | Demographics and clinical information obtained from self-reports and cancer registry  MOS-SSS  Perceived Personal Control Scale  Life Orientation Test-R. | SF-36 v2.0 – two subscales: physical component summary (PCS), mental component summary (MCS) | Coefficient of CAM found to be significantly associated with only MCS (not PCS) in linear regression adjusted for gender, age, race/ethnicity, marital status, education and optimism. However, debatable whether the difference between CAM users and non-users is clinically relevant (mean difference less than 10% of total scale). Perceived control fully mediated this relationship. |
| 7. | Hammersen et al., 2017 (Germany) [8] | To explore the impact of personal and health-related factors on sleep quality, and examine the relationship between sleep quality and HRQOL. | Cross-sectional correlational; t-test and regression analyses | 515 German HL and NHL survivors, all of whom were identified from a population-based cancer registry. Mean age = 63.1 years (SD – 14.4), and averaged 9 years post-diagnosis. Majority had NHL (77.3%, n = 398), underwent chemotherapy (83.4%, n = 403), and were married 67.6%). Race/ethnicity not reported. | PSQI  World Health Organisation-5 (WHO-5) wellbeing index (measured as a predictor of sleep quality)  Health perception measured via two statements: (1) whether participants felt cured, experienced a recurrence, metastases or other secondary malignant diseases, and (2) whether after malignant disease, patients felt an increased lack of energy, tiredness and inefficiency (measured as a predictor of sleep quality) | SF-36 v1 | High prevalence (51.8%) of survivors reported poor sleep quality.  Poor sleepers had significantly impaired PCS, MCS, and each dimension e.g., physical functioning, role-emotional, mental health) compared to good sleepers and norm.  Risk factors of sleep quality = depression, exhaustion, higher age, inability to work, endocrinological disorders, female gender.  NHL had lower scores in all HRQOL domains compared to HL survivors, whereby differences were significant for physical functioning and physical role functioning. |
| 8. | Husson et al., 2014 (The Netherlands) [9] | To examine relationship between perceived information provision at baseline and HRQOL, anxiety and depression among lymphoma patients after 2 years. | Prospective correlational; Multivariate linear regression | 355 participants completed questionnaire at T2 (1186 at T1). All between 6-months to 10 years post-diagnosis (mean = 4.5 years). Mean age of 60.7 years. Majority underwent chemotherapy (71%). Race/ethnicity not reported. | EORTC QLQ-INFO25  Time since diagnosis: <2 years vs. >2 years | EORTC QLQ-C30  HADS | Satisfaction provision found to be important for physical and emotional functioning (not anxiety/depression) of patients ≥ 2 years post-diagnosis (not <2 years) but this finding was not robust overtime. Even so, patients who were stably satisfied (i.e., satisfied at T1 and T2) had better mental HRQOL compared to patients who were unstably satisfied (T1 satisfied -> T2 unsatisfied OR T1 unsatisfied -> T2 satisfied) and stably unsatisfied. |
| 9. | Loge et al., 1997 (Norway) [10] | To assess the levels of psychological distress and identify predictors of anxiety/depression caseness after cancer cure. | Cross-sectional correlational; Multiple logistic regression. | 459 Norwegian HL survivors. Mean age at time of diagnosis 32 years (SD = 11.8). Majority had substage A disease (68%), had no relapse (93%), and were married/cohabitating (75%). Race/ethnicity not reported. | Sociodemographic and clinical characteristics from self-reports and cancer registry. Questionnaire included questions on sociodemographic status at time of diagnosis and at present time. Six questions asked about psychiatric symptoms (if experienced and type of dominating symptom), psychiatric treatment (psychiatrist/psychologist, medication, hospital/clinic), and consulting physician for psychiatric complaints before, during and after treatment. | HADS – Norwegian version | In univariate analyses: (1) Women, those separated/divorced, those poorly educated reported, those 7-10 yrs post-diagnosis, and those with psychiatric symptoms before HL or during treatment reported higher levels/cases of anxiety. (2) Those of older age, (50-59, >=60yrs), separated/divorced, poorly educated, had B-symptoms, 7-10 yrs post-diagnosis, and had psychiatric symptoms before HL or during treatment reported higher levels/cases of depression.  In multiple logistic regression: (1) Anxiety caseness predicted by those >= 7 yrs post-diagnosis, underwent irradiation and chemotherapy combined, had low education status, had psychiatric symptoms before HL or during treatment. (2) Depression caseness predicted by age and having psychiatric symptoms before HL. |
| 10. | Magyari et al., 2017 (Hungary) [11] | To evaluate frequency of psychological distress and its risk factors among HL survivors (in the context of work being a critical aspect of normal life) | Cross-sectional correlational; Chi-squared or Fisher's exact test (categorical variables), independent samples t-test, Mann-Whitney test, ANOVA, Kruskal-Wallis test (continuous variables), Spearman's correlation. | 140 HL survivors in Hungary who were of working age. Mean age at survey completion = 44.82 years, SD = 14.55. Mean age at time of diagnosis = 32.13 years, SD = 13.05. Race/ethnicity not reported. | Self-reported sociodemographic characteristics  Self-reported psychiatric treatment at time of diagnosis | HADS – Hungarian version  Perceived Stress Scale (PSS)  General Health Questionnaire (GHQ-12)  Dysfunctional Attitude Scale form A (DAS-A)  Sense of Coherence (SOC-13) – Hungarian version | HADS: 25% (34/140) had caseness scores (scoring >=8 or higher) with HADS anxiety 10% (14/140) with HADS depression where means for both HADS anxiety and depression were found to be significantly higher among inactive HL survivors.  Inactive HL survivors reported significantly higher means for perceived stress (PSS), DAS-total and DAS perfection (only DAS dependency difference was not significant).  10% (14/140) HL survivors had abnormal levels of distress in GHQ - 22% of inactive participants had abnormal GHQ compared to 4% of active participants.  Active survivors had significantly better coping (higher SOC mean score) compared to inactive survivors. |
| 11. | Mariegaard et al., 2021 (Denmark) [12] | To investigate prevalence of self-reported cognitive impairment and its relation to illness and treatment characteristics and mental health in HL and DLBCL survivors | Cross-sectional correlational; Chi-square test and post-hoc analyses (categorical variables), Mann-Whitney U Test (continuous variables), and Cochran-Armitage. | 115 HL and DLBCL survivors ≥ 6 months post-remission (mean = 29.6 months post-treatment). Mean age 40.3 years, SD = 11.7, range = 20 – 60 years. Race/ethnicity not reported. | Self-reported psychosocial and sociodemographic information (e.g., educational attainment, previous neurological illness, previous treatment for psychiatric illness, weekly alcohol consumption, previous drug use)  Clinical characteristics obtained from medical records  Behaviour Rating Inventory of Executive Function-Adult (BRIEF-A) – Danish version.  PSQI – Danish version, whereby PSQI > 5 = poor sleep quality | HADS – Danish version. Cut off score for caseness is > 11.  EORTC QLQ-C30  PSS | Impaired executive functioning associated with greater symptoms of depression, anxiety, stress, poor sleep quality and lower quality of life.  Clinical levels of anxiety symptoms were present in 29% of survivors who reported impaired executive functioning vs. 3% of survivors not reporting impairment.  Could not compare two groups on depression due to small number of clinical depression cases, but clinical levels of depression were present in 7% of survivors reporting impairment vs. none in survivors who did not report impairment.  Poor sleep quality had a prevalence of 80% in survivors reporting impairment compared to 36% of survivors who did not report impairment. |
| 12. | Matasar et al., 2015 (USA) [13] | To assess the late morbidity and mortality of adults treated with first line trial for HL over a 25-year period | Prospective correlational; Wilcoxon Rank Sum Test, Kruskal-Wallis test | 238 HL patients treated with first-line trial. Median age 49 years, ranging 25-88 years. Majority were white (96.6%), non-Hispanic (95.8%), and married/living with partner (80.7%), | Sociodemographic and clinical characteristics obtained via self-report and confirmed via chart review | Functional Assessment of Chronical Illness Therapy – Fatigue  Fears of Recurrence Questionnaire (FRQ)  HADS  SF-12 (relevant questions) | Borderline and major anxiety more common in women (P = .002) and in patients who were younger at times of treatment and survey (each, P = .01).  No differences noted by age, gender, time from treatment, or treatment exposure in the case of depression.  Self-reported psychiatric morbidity (i.e., subjective depression, anxiety, other psychiatric illness, or multiple psychiatric morbidities requiring psychotherapy or medical therapy) associated with higher radiation therapy doses and ppl who were of younger age at treatment.  FCR associated with younger age at time of survey and higher doses of radiation. |
| 13. | Oerlemans et al., 2014 (The Netherlands) [14] | To (1) compare the prevalence of anxiety/depression with normative population, (2) assess prevalence of anxiety/depression over time and identify subgroups of patients, (3) assess impact of anxiety/depression on HRQOL | Prospective case control; Linear mixed-effects models | 489 patients with HL (n = 180) and DLBCL (n = 309) ranging 0-10 years post-diagnosis. At T1: mean age for HL patients = 46.1 years; mean age for DLBCL patients = 63.6 years.  360 HL and 425 DLBCL age- and sex-matched controls.  Race/ethnicity not reported. | Self-administered comorbidity questionnaire (SCQ)  Sociodemographic characteristics obtained from self-reports (i.e.,marital status, educational level) and cancer registry (background characteristics e.g., gender, date of birth).  Clinical characteristics obtained from cancer registry | HADS  EORTC QLQ-C30 | Comorbid diseases and lower education levels identified as longitudinal risk factors for anxiety/depression in both HL/DLBCL patients. Age may be possible risk factor - in DLBCL patients, younger age associated with greater anxiety whereas older age associated with greater depression. In HL patients, younger women and older men reported greater levels of depression. |
| 14. | Øvlisen et al., 2022 (Denmark) [15] | To investigate the risk of mental health complications in various subtypes of NHLs compared to sex-, age-, and country-matched controls. | Prospective case control; Cox-proportional hazards regression analyses, Gray’s test, marginal Cox proportional hazards models, landmark analysis | 8750 Danish NHL patients, followed from diagnosis to end of follow-up. Median follow-up was 7.1 years. Median age = 66 years, interquartile range = 57-75 years.  43750 controls matched by age, sex, and country of origin.  Race/ethnicity not reported. | Sociodemographic and clinical characteristics obtained via multiple registers including Danish Civil Registration System, Danish Lymphoma Registry, Danish National Patient Registry, Danish Education Register. | Registries used to obtain the following:   - use of psychotropic drugs (first and second prescription) - first in-patient/out-patient visit at psychiatry department - completed suicide or first incident of intentional self-harm | Across most NHL subtypes, risk of PD prescriptions higher in patients >70 years, CCI ≥ 1, ECOG performance score >1, and higher IPI (for aggressive lymphomas) whereas higher educational level was associated with lower risk of PD prescriptions.  Patients with relapse/progression had higher two-year cumulative use of psychotropic drugs compared to matched comparators - relapsed DLBCL (20.8%), aggressive T-cell NHL (25%), and other aggressive B-cell NHL (26.3%) showed highest cumulative incidence.  NHL patients were not more likely than matched comparators in psychiatric visits.  Completed suicide and intentional self-harm significantly higher in NHL patients compared to matched comparators. However, authors did highlight that absolute risk increase here was minimal (0.3% NHL patients vs. 0.2% comparators). |
| 15. | Øvlisen et al., 2020 (Denmark) [16] | (1) To compare cumulative incidence of depression and anxiety between Danish patients with HL and matched population and (2) analyse if potential initial increase in depression/anxiety after diagnosis would normalise over time | Retrospective case-control; 5-year cumulative incidence (Aalen-Johansen estimator), adjusted Cox regression analyses | 945 Danish patients with HL, followed from diagnosis (median follow-up 7.2 years). Median age = 39 years, ranging 18-92 years.  4275 matched controls with no prescription of psychotropic drugs within 10 years prior to inclusion date of patient  Race/ethnicity not reported. | As above | National Prescription Registry used to obtain prescription of psychotropic drugs (antidepressants, antipsychotics and anxiolytics) | Risk factors of high 5-year cumulative incidence (measured from baseline) include older age, advanced disease stage, CCI score >1, poor ECOG performance status (>1) and patients treated with 6-8 cycles of ABVD. Similar to healthy population, CCI >1 was a risk factor and education level was associated with PD use. Unlike healthy population, sex was not associated with PD use (healthy population -> females were more likely to be prescribed PD) and younger threshold (>30 years vs. >60 years) was a risk factor in HL patients. |
| 16. | Pettengell et al., 2008 (UK) [17] | To determine relationship between disease states and health functioning in follicular lymphoma patients | Cross-sectional correlational; Kruskal-Wallis, Ordinary least-squares linear regression, Mann-Whitney U-test | 222 follicular lymphoma patients across 5 survivorship stages. Mean age was 60.4 years, ranging from 36-85 years. Race/ethnicity not reported. | Disease stages: newly diagnosed, relapsed, partial response, complete response/remission (at first follow-up), disease-free (no detectable disease beyond first follow-up  Sociodemographic characteristics obtained via self-report; clinical characteristics obtained via medical records | Function Assessment of Chronic Illness Therapy-general and additional lymphoma subscale (FACT-LYM)  Work Productivity and Activity Impairment Scale (WPAI)  HADS | Difference found between the five disease groupings in FACT-LYM (except social wellbeing), HADS and activity impairment. Relapsed group reported lowest mean scores compared to other groups and this was supported by linear regressions  Remission/complete response group predictive of greater anxiety but not depression  Participants receiving chemotherapy had worse health functioning, depressive symptoms and activity impairment compared to those not receiving chemotherapy |
| 17. | Poe et al., 2012 (USA) [18] | To explore treatment decision-making and distress in individuals previously diagnosed with follicular lymphoma | Cross-sectional correlational; Descriptive statistics | 32 follicular lymphoma survivors, majority of whom were in remission (75%), female (59%), and non-Hispanic white (94%). Mean age was 58 years, SD = 10.5, range = 36-78 years. | Role of others in decision-making: Decision resource checklist, Importance of Significant Others, Control Preferences Scale  Decisional outcomes: Decisional Conflict Scale (DCS), Decision Regret Scale (DRS), Satisfaction with Decision Scale (SWD) | Revised Impact of Event Scale  HADS | Most participants preferred to collaborate with healthcare professionals in their treatment decisions and actively consider the opinions of their friends/families. Participants seemed largely satisfied and unconflicted about their recent treatment decisions, suggesting that other things may contribute to their experienced distress apart from engaging in shared treatment decisions. However, authors noted the possibility that participants may have become familiar with weighing treatment options as time passed - the first treatment decision following diagnosis may induce more conflict and regret. |
| 18. | Posluszny et al., 2016 (USA) [19] | To examine existential challenges experienced by cancer survivors | Cross-sectional correlational; Multivariate linear and logistic regressions | 429 US lymphoma survivors (43% HL, 57% NHL) averaging 7-years post-diagnosis. Mean age of 44 years. Predominantly White (87.1%). Subset from a 2010 LIVESTRONG survey study of 4286 participants. | Self-reported sociodemographic and clinical characteristics | Existential challenges:   1. Subjective meaning associated with disease: Constructed Meaning Scale 2. Cancer worry: (1) preoccupation with concerns about cancer, (2) worries about dying from cancer, (3) worries about cancer coming back 3. Sense of security: single-item measure 4. Sense of identity: single-item measure 5. Grief: single-item measure 6. Guilt: single-item measure | Factors highlighted:   1. Marital status: Being unmarried associated with lower Constructed Meaning and higher odds of reporting identity concerns 2. Gender: Women more likely to report concerns of grief and guilt. 3. Stem cell transplant: Participants receiving stem cell transplantation reported lower levels of meaning and more concerns around security and identity. 4. Age: Older respondents worried less about cancer. 5. Time since diagnosis: Longer time since diagnosis associated with less cancer worry but more grief and guilt over deaths of other survivors. |
| 19. | Roper et al., 2013 (USA) [20] | To describe changes in HRQOL and to identify supportive care services used after treatment for HL in young adults | Prospective repeated measures descriptive; Non-parametric Friedman tests and Wilcoxon Signed Rank tests (based on Bonferroni correction of 0.0167). | 40 young adults with HL completed survey at baseline. Mean of 30.9 years, SD of 5.8 years, ranging 27-40 years. 38 participants completed surveys at all timepoints – demographics not reported. Majority were female (60%), married (63%), Caucasian (90%), lived with children (55%), employed or earning income within last 12 months (88%), had stage I or stage II disease (83%), and received chemotherapy only (58%) | Demographic and supportive care services: self-reported  Clinical characteristics: medical chart review  Time: collected data at 4 timepoints: towards end of treatment (baseline) and one-, three-, and six-months post-treatment. | Physical symptom distress: Symptom Distress Scale (SDS)  Emotional distress: HADS  Intimate relationships: social and relationship scale in Impact of Cancer (IOC)  Functional status: physical health items in SF-12 | Mean scores for depression and anxiety were all within normal range and improved significantly from baseline to six months  Depression scores improved significantly from baseline to one month (p = .010) but there were no significant improvement in anxiety levels (p = .029 - based on Bonferroni correction levels of 0.0167)  Prevalence of moderate/severe levels of HADS reduced overtime – 12.5% had severe emotional distress/anxiety at baseline, and this decreased to 7.9% by 6 months after treatment. Although incidence of severe anxiety decreased to 2.6% at 1 month, it continued to increase to 5.4% at 3 months and slightly more at 6 months  Physical distress also significantly improved at all other timepoints compared to baseline. Also, use of supportive care services seemed to decrease overtime. However, interesting to note that as economic inquiries declined at 6 months, use of health behaviour programs, especially fitness programs, increased at 6 months |
| 20. | Smith et al., 2008 (USA) [21] | To examine prevalence of PTSD symptoms in adult NHL survivors and identify risk factors associated with PTSD symptoms | Cross-sectional correlational; Multiple linear and logistic regressions | 886 NHL survivors ≥ 2 years post-diagnosis averaging 10.2 years post-diagnosis (range: 2-44 years). Mean age of 63 years at study enrolment (SD = 13.5), ranging 25-92 years. 46% aged ≥ 65 years. Mean age of 53 years at diagnosis (14.2 years), ranging 19-87 years. Majority were White (85.5%), married (73.1%), receiving chemotherapy (78%) and were not in treatment for NHL (85%). | Demographic information: self-reported  Clinical data: tumour registry databases  Health-related problems:   1. Self-Administered Comorbidity Questionnaire 2. Relevant questions about health care use and secondary cancer status adapted from Childhood Cancer Survivor Study survey   Psychosocial aspects:   1. Perceived availability of social support: MOS-SSS 2. Cancer appraisal: ALTTIQ 3. Employment and insurance-related issues: 24-item instrument developed by CALGB clinical research group | PTSD Checklist (PCL-C) – Civilian Version. PTSD identified via cut-off score of ≥ 44 or scoring of (1) ≥ 3 for one or more of re-experiencing symptoms, (2) ≥ 3 for one or more avoidance symptoms, and (3) ≥ 2 or more arousal symptoms. | More recent diagnosis, greater comorbidity, less social support, more negative appraisals, more employment and insurance issues, and less education were linked to worse PCL-C scores and greater odds of reporting partial or full PTSD.  Younger age and having active disease was associated with PCL-C scores (linear regression) but not partial/full PTSD (logistic regression),  Non-White race predicted greater PCL-C scores (linear regression), and was marginally significant as a predictor of partial/full PTSD (p = .07) (logistic regression).  In linear regression, the above factors explained 38.5% of the variance in PCL-C scores. |
| 21. | Smith et al., 2010 (USA) [22] | To assess contribution of survivors' perception of positive and negative impacts of cancer to explain QOL-related outcomes | Cross-sectional correlational; Hierarchical multiple linear regressions | 652 NHL survivors (subset from Smith et al., 2008 study) ≥ 2 years post-diagnosis averaging 10.8 years post-diagnosis (SD = 7.5). Mean age at study enrolment of 62.7 years (SD = 13.5). Mean age at diagnosis of 51.9 years (SD = 14.2). Majority were Caucasian (88%) and close to half (46%) were older adults ≥ 65 years. | Demographic, clinical characteristics including comorbidity: self-reported in survey including Self-administered Comorbidity Questionnaire  Psychosocial: (1) MOS-SSS, (2) ALTTIQ, (3) 24-item instrument developed by CALGB clinical research group  Impact of cancer: IOCv2 | Health status: mental component summary (MCS) and physical component summary (PCS) in SF-36  QOL: FACT-G | IOC is associated with health status and QOL. Many relationships between characteristics and QOL/health status became non-significant after addition of IOC.  In model predicting MCS, only age, comorbidity and social support remained significant predictors after addition of IOC.  In model predicting FACT-G, only ethnicity, education level, employment status, age, comorbidity, and social support remained significant after addition of IOC. |
| 22. | Soares et al., 2013 (Brazil) [23] | To assess association of social network and social support with QoL and fatigue in long-term HL survivors | Cross-sectional correlational; Mann-Whitney test, linear regression | 200 long-term HL survivors with median of 7 years post-diagnosis, ranging 3.6-12.7 years. Median age of 29 years, ranging 16-77 years. Majority received ABVD chemotherapy (92%) and had a lower socioeconomic status (77%). Race/ethnicity not reported. | Demographic and clinical characteristics: Self-reported with instrument commonly used in publicity/political polls in Brazil  Perceived availability of social support: MOS-SSS | Health status: SF-12  Cancer-specific QOL: QOL-CS questionnaire  Fatigue: Multidimensional Fatigue Inventory (MFI) (Brazilian Portugese-translated) | Larger number of friends/relative associated with better SF-12 scores for pain, social functioning, mental health, emotional role, and better overall score in QOL-CS. Affective support associated with all dimensions of QoL and fatigue.  Male, younger age (< 45 years), higher SES and higher educational level had better scores on physical domains of QoL. Males and participants <45 years also had lower scores on fatigue scale (general fatigue and reduced activity respectively). Males had higher score in social functioning and mental health QoL. Patients who received radiation therapy had higher scores in social functioning and mental health QoL, but as they had localised disease at diagnosis, authors stated that no conclusions should be drawn from this observation. |
| 23. | Spector et al., 2015 (USA) [24] | To determine whether NHL survivors are meeting health-related guidelines, and examine relationships between these lifestyle factors and HRQOL/post-traumatic stress | Cross-sectional correlational; Multiple linear regression | 566 NHL survivors averaging 15.2 years post-diagnosis, with majority not receiving active treatment (93%) and have not had a recurrence within the past 5 years (75%). Mean age of 67.2 years (SD = 12.5 years). Majority were white (87%), married or living with partner (77%), retired (62%), and received less than a college education (57%). Participants were a subset from from Smith and colleagues’ survey study [25]. | Demographic and clinical characteristics: Self-reported questionnaire including Self-Administered Comorbidity Questionnaire (SCQ)  Meeting health recommendations by the American Cancer Society: being a non-smoker, ≥ 150 minutes of moderate-intensity aerobic exercise, ≥ 5 servings of fruit and vegetables each day, having a BMI of < 25kg/m^2^  Physical activity: number of times/week and minutes/time participants exercised at moderate-intensity in a typical week)  Fruit and vegetable intake: servings of vegetables usually eaten/drank in a day  Tobacco use: question from CDC and Prevention Behavioral Risk Factor Surveillance System – “have you smoked at least 100 cigarettes?” “do you now smoke cigarettes every day, some days, or not at all?”  BMI: derived from self-reported height and weight) | QOL: SF-36  PTSD: PCL-C | Participants who met 3 (25%) or 4 (11%) of health guidelines had better HRQOL and less stress compared to those meeting fewer recommendations.  Better mental HRQOL was associated with meeting all 4 recommendations (b = .47), being a non-smoker (b = .71), and meeting physical activity recommendations (b = .41). Lower PTS scores were associated with these three factors at b = -.41, b = -.73, and b = -.23 respectively.  Meeting fruit/vegetables intake recommendations associated with better mental HRQOL (b = .18).  BMI <25 kg/m^2 may be associated with lower mental HRQOL.  Similar factors (i.e., smoking, exercise, meeting all 4 healthy recommendations) associated with physical HRQOL.  Being white associated with being more likely to meet all 4 recommendations. Females and participants with higher education and income more likely to engage in greater number of health behaviours. |
| 24. | Tilch et al., 2022 (Germany) [26] | To explore the incidence of depression and anxiety diagnoses in lymphoma patients compared to patients without cancer over a 10-year timeframe | Retrospective cohort; Cox regression analyses | 4817 lymphoma patients in Germany (687 with HL, 4130 with NHL) followed from diagnosis to 10-years post-diagnosis. HL patients had a mean age of 49.8 years (SD = 18.2) whereas NHL patients had a mean age of 61.9 years (SD = 16.7).  Controls: 4817 age-, sex-, index-year-, yearly-consultation-matched controls without cancer  Race/ethnicity not reported. | Demographic and clinical characteristics: Disease Analyzer database (IQVIA) from general practitioners  Lymphoma diagnosis: Comparison of outcomes between people with and without lymphoma. | Anxiety and depression: diagnoses of anxiety and depression obtained from IQVIA | HL and NHL were linked to increased incidence of depression compared to matched non-cancer controls, even when age and gender were accounted for.  NHL was associated with incidence of anxiety disorders, especially in men. |
| 25. | Troy et al., 2019 (USA) [27] | To describe the magnitude and sources of patient-reported distress | Retrospective cohort; Generalised Estimating Equations | 304 HL patients at various stages in survivorship period. Median age of 34 years at diagnosis, ranging from 6-80 years. Majority were White (62.3%), non-Hispanic or Latino (84.9%), had stage I/II disease (56%) and received chemotherapy only (72%). | Sociodemographic and clinical characteristics: Abstracted from medical records  Survivorship status: Active treatment, surviving <5 years, or surviving ≥ 5 years since diagnosis | Distress: abstracted data from NCCN DT and PL assessed as part of routine care. DT score ≥ 4 regarded as “actionable distress”. | Distress burden is low on average, but actionable distress frequently reported across clinical encounters, with distress being most frequently reported by patients on treatment (32.5% of their total visits) compared to survivors (<5 years: 20.4% and ≥ 5 years: 28.7% of their total visits).  Disease-related prognostic factors (e.g., age, sex, pathologic subtype, stage at diagnosis) were not associated with actionable distress.  Across survivorship periods, pattern in frequency of problem domains remains the same: physical most frequently reported (especially fatigue), then emotional, practical, and family/spiritual. Fatigue associated with depression and other psychological symptoms such as nervousness, sadness, fear, and loss of interest |
| 26. | Varela et al., 2013 (USA) [28] | To examine the prevalence of full and partial PTSD in long-term HL survivors, and explore the clinical relevance of partial PTSD by assessing functional impairment associated with sub-threshold symptomatology | Cross-sectional case-control; Fisher’s Exact Tests | 105 long-term HL survivors with a median of 16 years post-diagnosis (ranging 7-34 years). Median age at participation of 43 years, ranging 24-71 years. Median age at diagnosis = 25 years, range = 6-61 years. Majority were White (98.5%), received radiotherapy (96.2%), had stage IIA cancer (60%), and completed training after high school (92.3%). 25.7% experienced relapse.  101 matched controls (siblings) from previously established cohort [29] Median of 42 years in age. | Demographic and medical characteristics obtained from previous study database (Ng et al., 2005). | Posttraumatic Diagnostic Scale (PDS) – full PTSD defined by DSM-IV-TR standards and previously published PDS criteria; partial PTSD defined by previously published criteria | **Females may be predisposed to experience full PTSD, whereas both males and females may experience partial-PTSD.** No significant difference between survivors and sibling controls in regards to prevalence of full PTSD. A greater number of female survivors (16.9%) reported full PTSD compared to female sibling controls (5.5%), although this difference was only approaching statistical significance (p = .057). However, when comparing for partial-PTSD, a greater proportion of survivors (35.2%) met this criterion compared to siblings (17.8%), p = .004. A higher number of male/female survivors met this criterion compared to male/female siblings, p = .04.    **Experience of cancer should not be assumed to be the most traumatic event for all cancer patients.** Some survivors (12.4%) did not endorse having had life-threatening illness as a traumatic event, and these survivors did not have significant sociodemographic/clinical differences (i.e., age, gender, age at diagnosis, stage of disease, radiation therapy or chemotherapy received) from survivors who reported having experienced a life-threatening disease as traumatic. All 14 cancer survivors who reported full PTSD endorsed “life-threatening illness” as a traumatic event but more than half reported that a non-cancer trauma (e.g., car accident, other disease/treatment procedures, physical abuse) was their worst experienced trauma. |
| 27. | Walburg et al., 2019 (France) [30] | To measure the prevalence of fear of cancer recurrence in French lymphoma survivors and determine factors associated with clinical levels of fear of cancer recurrence | Cross-sectional correlational; Multivariable analysis (type of regression not indicated) | 108 French HL and NHL survivors in first three-years of disease-free survival (averaging 1.6 years since complete remission). Mean age at diagnosis of 51.8 years, SD = 14.7 years. All participants had previously participated in Ambulatory Medical Assistance – After Cancer (AMA-AC) program. Race/ethnicity not reported. | Sociodemographic and clinical characteristics: abstracted from medical records at baseline (i including CCI  Anxiety and depression: HADS (French version)  HRQOL: SF-36 (French version) | Fear of cancer recurrence: Fear of Cancer Recurrence Inventory – Short Form (FCRI-SF). Cut-off score of ≥ 13. | Factors highlighted (controlled for gender, age, occupational status, cancer type, time since complete remission):   - Higher depression and anxiety scores (HADS) at baseline associated with greater likelihood of experiencing clinical levels of fear of cancer recurrence - Later time since complete remission: Participants at 6 months and 36 months reported greatest mean score for fear of cancer recurrence. Participants at 3 months reported the lowest mean scores. - Professional working activity prior to diagnosis and higher depression/anxiety scores at baseline were associated with clinical levels of FCR   Age, gender, type of lymphoma, mental and behavioural disorders or other comorbidities were not significant predictors of clinical levels of fear of cancer recurrence. |
| 28. | Wang et al., 2013 (China) [31] | To (1) assess levels and sources of distress in lymphoma patients (2) examine factors of level of distress, and (3) evaluate cut-off score of DT against HADS | Cross-sectional; Multiple logistic regression | 323 Chinese lymphoma patients ≤ 1 month to > 12 months post-diagnosis. Mean age of 45 years (SD = 16.2). | Demographic and clinical information: self-reported | Distress: DT and PL (Chinese version). Cut-off score for clinically significant distress was ≥ 5.  Anxiety and depression: HADS (Chinese version). HADS-Anxiety and HADS-Depression score ≥ 9 = caseness | B-symptoms found to be robust predictor of greater DT and HADS scores.  Participants with emotional and physical problems (PL) more likely to report clinically significant distress. Other problems (i.e., practical, family, spiritual/religious problems) were not found to be significant predictors.  Age, gender, treatment, marital status, diagnosis, education not significantly correlated with DT or HADS score cut-offs.  Disease stage in patients' opinions and interval from time of diagnosis were significantly associated with continuous scores of DT and HADS but not cut-off scores. |

Note: Summary of 28 quantitative studies reviewed, ordered alphabetically. Abbreviations: HL = Hodgkin Lymphoma, NHL = Non-Hodgkin Lymphoma, DLBCL = Diffuse large B cell lymphoma, MOS-SSS = Medical Outcomes Study Social Support Survey, SF-36 = 36-item short-form health survey, HRQOL = health-related quality of life, CALGB = Cancer and Leukemia Group B, SD = standard deviation, ANOVA = analysis of variance, MANOVA = multivariate analysis of variance, MANCOVA = multivariate analysis of covariance, PTSD = post-traumatic stress disorder, EORTC QLQ-C30 = European Organization for the Research and Treatment of Cancer Quality of Life Questionnaire, HADS = Hospital Anxiety and Depression Scale, PSQI = Pittsburgh Sleep Quality Index, EORTC QLQ-INFO25 = European Organization for the Research and Treatment of Cancer Quality of Life Information Module, CCI = Charlson Comorbidity Index, ECOG = Eastern Cooperative Oncology Group, IPI = International Prognostic Index, SF-12 = 12-item Short Form Survey, ALTTIQ = Appraisal of Life Threat and Treatment Intensity Questionnaire, FACT-G = Functional Assessment of Cancer Therapy – General, QOL-CS = Quality of Life-Cancer Survivors, b = beta, BMI = body mass index, NCCN = National Comprehensive Cancer Network, DT = Distress Thermometer, PL = Problem List.

**Table 2**

*Summary of Qualitative Studies*

| 1. | Chircop & Scerri, 2018 (UK) [32] | To identify frequencies at which metaphors were used by participants, which aspects of the participants' experiences were described using metaphors, and whether metaphors were used to empower or disempower the participants | Secondary analysis of phenomenological study (not cited); Pragglejaz metaphor analysis | 6 adult NHL patients undergoing chemotherapy. Mean age = 63 years. All participants were in stable relationships. Race/ethnicity not reported. | Original study involved individual semi-structured interviews lasting 60-90 mins. | Participants described experience as "war" (shell-shocked, battlefield), "prison" (lower immunity meant being restricted to own hospital rooms, felt trapped being unable to resume daily activities, one participant hoped for freedom after end of treatment), "journey" (long, uncertain road, expressed desire to depart current situation by committing suicide)  Use of metaphors may be empowering or disempowering, depending on the individual.  Participants felt powerless and distressed by fatigue, describing it as feeling "trapped" in their own bodies and homes |
| --- | --- | --- | --- | --- | --- | --- |
| 2. | Friesen-Storms et al., 2021 (The Netherlands) [33] | To understand the experiences of lymphoma survivors in early aftercare who have received an aftercare consultation based on evidence-based guidelines | Cross-sectional qualitative; Thematic narrative analysis | 22 lymphoma survivors (and 9 partners, demographics unknown) in early aftercare (3-8 weeks post-treatment). Median age = 65.5 years, range = 27-74 years. Majority of participants were male (63.64%). 95.45% were diagnosed with NHL, 4.55% were diagnosed with HL. 18.18% had ≥ 2 recurrences. 86.36% received chemotherapy alone, 9.09% received chemotherapy and biological therapy, and 4.55% received chemotherapy and bone marrow transplant. Race/ethnicity not reported. | Narrative interviews encouraging participants to tell their experiences of early aftercare in personal stories. Example questions include “How did you resume your everyday life after you received the diagnosis and treatment?”, after which participants were prompted to describe their experiences with the care received and emerging topics were discussed. | Participants described how cognitive functioning and hair loss contributed to distress. Possible financial impact and considerations involved in cancer was also described, although how this relates to distress was less explicit. Moreover, participants stressed the importance of resuming pre-diagnosis activities (e.g., work, walking, cycling). Social support was highly valued and participants stressed the importance of involving social support networks in patient conversations.  For patients who were treated after a recurrence and had a more chronic form of lymphoma, uncertainty often remained a constant part of their life. Some of these patients felt that taking control by planning euthanasia reduced the feeling of uncertainty.  Highlighted importance of holistic understanding of patients – other life events (e.g., severe comorbidities, death of partners/relatives, problems with children/co-parenting issues) may be an additional burden to patients; on the other hand, having experienced tough life events may facilitate patients in dealing with challenges of cancer.  Importance of support from healthcare professionals was stressed. Components that patients appreciated include continuity of care, being provided with sufficient information, personal attention and empathy. Aftercare consultation was appreciated as it focused on psychosocial aspect of care and highlighted next steps after treatment |
| 3. | Glasdam et al., 2020 (Sweden) [34] | To explore how patients with malignant lymphoma who underwent curative treatment cope with cancer in everyday life (understood through Antonovsky's theory of sense of coherence). | Cross-sectional qualitative; Latent thematic analysis | 9 malignant lymphoma survivors (7 men, 2 women) who completed chemotherapy 4-18 months prior to study interview. Mean age = 65 years, SD not reported, range = 35-84 years. 3 patients returned to work with reduced working hours and other 6 have retired. 7 were married, 1 single, 1 a cohabitant. Race/ethnicity not reported. | Semi structured interviews guided by Antonovsky's theory of sense of coherence which asked about (1) short life history with focus on social background, (2) everyday life under chemotherapy, (3) everyday life today after completed treatment | Prior life experiences and how patients coped with them may inform how they cope with treatment. Coping strategies highlighted: reframing, seeking control/projectification, diversion/seeking sense of normality  Transition from treatment to post-treatment highlighted as difficult time for many patients due to lack of comprehensibility.  Social support nuanced – some family members/friends had an active function (reinforced patients’ own resources, validated patients’ situations) and patients were able to expand their circle of friends (other patients). However, other loved ones were put on standby such that during treatment, social interactions were avoided and this resumed as normal after treatment. Yet other loved ones disappeared, leaving patients feeling alone and disappointed |
| 4. | Hackett & Dowling, 2019 (Ireland) [35] | To explore survivors' experiences at end of treatment and follow-up care | Cross-sectional qualitative; Thematic analysis | 14 lymphoma patients. Age at diagnosis: 3 were aged 18-39, 5 aged 40-64, and 6 aged >65. Majority (64.3%, n = 9) underwent chemotherapy-only. Participants were 3-60 months post-treatment prior to study commencement. Race/ethnicity not reported. | Semi-structured interviews asking about (1) feelings once active treatment was complete, (2) feelings and opinions regarding challenges once treatment was complete, (3) knowledge regarding end of treatment visit and information received, (4) Participants' views on survivorship care plan | Transition period between end of treatment to start of survivorship may be abrupt and traumatic for some patients (described as lonely, loss of security, unprepared, disappointed), especially those who had previously experienced intensive primary treatment.  Fear of cancer recurrence intensified leading up to follow-up visits whereby it was most intense in first two years post-treatment but reduced with time. This was less intense for individuals who experienced recurrence for indolent lymphoma  Relationships were reported to strengthen and deepen, and this was greatly valued by participants. However, some relationships were lost as friend and family were unsure how to deal with diagnosis. Companionship with other patients were also greatly valued, but this was not available in the post-treatment period and only 2 participants shared experiences of accessing cancer support groups.  Being able to return to work was highly valued, especially for patients who were employed prior to diagnosis - provided indication of going back to normal life. Some participants have also linked it to a source of emotional support.  Several physical symptoms were also highlighted: fatigue ("affected ability to socialise, function at work or spend time with family"), recurrent infections ("'prolonged recovery time... source of anxiety and worry of recurrence"), weight gain (no mentions of how this links to distress) |
| 5. | Johansson et al., 2010 (UK) [36] | To explore occurrence of symptoms and relationships between them as perceived by patients with lymphoma before, during, and 14 months after the beginning of treatment. | Longitudinal qualitative; Interpretive description, grounded approach | 10 adult lymphoma patients recruited at treatment initiation. Mean age 45 years, ranging from 18-64 years. Race/ethnicity not reported. | Individual semi-structured interviews at three time-points after beginning of treatment:  T1 = 15 days  T2 = 112 days  T3 = 413 days.  Interviews were focused on exploring symptom experience (i.e., perception and response to symptom occurrence and symptom distress), with all interviews focusing on descriptions of symptom experiences up until time of interview and T1 interview beginning with invitation to describe health changes leading to diagnosis | Found that night sweats were highly interrelated with other symptoms,  Itching/pain may contribute to irritability and sadness. Social impact of itching/pain highlighted.  Interrelationships varied considerably by individual and timepoint.  Worrying and sadness were frequently reported across three timepoints. Also highlighted irritability as other form of emotional distress often linked to somatic issues that should be further explored. |
| 6. | Matheson et al., 2016 (UK) [37] | To explore experiences of young adults with HL and their psychosocial adjustment during first year following end of initial treatment - informed by authors' grounded theory of psychosocial adjustment to cancer | Longitudinal qualitative; Social constructivist grounded theory | 10 HL survivors 2- and 7-months post-treatment. Age ranged from 21-39 years, central tendency not reported. Majority White British (90%). | Individual semi structured interviews at 2- and 7-months post-treatment. Includes the following questions: (1) Can you tell me about your experience of completing cancer treatment, (2) Can you tell me about the impact of cancer on different areas of your life since finishing treatment? (3) Are there any areas where you felt you wanted more information or support?, (4) What do you think might help young adults in the future? | Factors highlighted:   1. appraisal/reframing (holding on to positive perceptions of and gains/advantages of YA cancer experience) 2. social/peer support (emotional support provided by existing friendships and other young cancer survivors helped to normalise experience and reduce perceived threats of disease) 3. unmet information needs (sense of uncertainty on how to deal with symptoms and psychosocial needs after perceived abrupt end of professional care after treatment) 4. physical symptoms (appearance changes, temporary dependence, and difficulty returning to pre-cancer physical activities may contribute to distress) |
| 7. | Monterosso et al., 2017 (Australia) [38] | To explore the post-treatment experiences and preferences for follow-up support of lymphoma survivors. | Cross-sectional qualitative; Thematic content analysis | 17 HL and NHL patients 6-30 months post-treatment. Mean age 63.8 years, ranging from 27-85 years. Race/ethnicity not reported. | Focus group semi-structured interviews which prompted exploration of informational, psychological, emotional, social, practical, physical and spiritual aspects of post-treatment support need. Participants were also asked to talk about what they thought would have been or could be of help to them in the post-treatment period. | Highlighted importance of social support (family, friends, peer support groups) and good clinician-patient relationships, although nuances were reported. Lack of information and preparation prior to completion of treatment seem to contribute to sense of uncertainty, fear, and loss. |
| 8. | Payne et al., 2019 (USA) [39] | To examine patients' and carers' care needs and research priorities. | Cross-sectional qualitative; Thematic analysis | Phase 1: 15 HL and NHL survivors and caregivers. Majority were White (46.7%).  Phase 2: 19 HL and NHL survivors and caregivers; median age of 63 years, ranging 21-80 years. Majority were White (89.5%). | Phase 1: Focus groups asking questions around (1) research priorities, (2) clinical care team, (3) interest in research, (4) survivorship, (5) barriers.  Phase 2: Individual interviews asking questions derived from focus group results, literature review and authors' expertise. | Factors highlighted/implied:   1. unmet information needs (e.g., patients reported difficulty obtaining clinical information around diagnosis, research opportunities, treatment decisions) 2. relatedly, clinical care team's lack of guidance and occasional unsympathetic attitudes may feed into fear of unknown;   social support as coping strategy to combat fear of unknown and anxiety, and it seemed that cancer diagnosis were perceived to strengthen social support networks (patients turned to family and friends for support emotionally, financially, physically), although there were also mentions that strains may arise from feeling burdensome to their support networks |
| 9. | Ruan et al., 2019 (China) [40] | To explore the illness experiences of lymphoma patients in China and describe the impacts of this disease on the everyday lives of these individuals | Cross-sectional qualitative; Conventional content analysis | 16 Chinese lymphoma patients across survivorship periods. Mean age of 41.4 years, ranging from 20-73 years. Majority had NHL (81.3%), were male (56.3%), married (75%), received tertiary education or higher (62.5%), had no religion (68.8%), and received chemotherapy only (75%). Race/ethnicity not reported. | Individual semi-structured interviews regarding (1) illness experiences since being diagnosed, (2) experiences when lymphoma was in state of remission, (3) experiences when lymphoma was in state of relapse, (4) how daily life was affected by lymphoma, and (5) negative changes experiences since diagnosis. | The belief that cancer was fated and concern for the integrity of the family facilitated acceptance of cancer.  (1) Changes in physical appearance, (2) public attitudes towards cancer and death (“ominous symbol”), and (3) impaired ability to work and care for their families contributed to negative self-image, which in turn influenced interpersonal relationships and career development. |
| 10. | Swash et al., 2018 (UK) [41] | To investigate NHL patients' (1) experiences of psychosocial needs, (2) why specific needs were felt to be important, and (3) the impact that these unmet needs had on overall patient experience | Cros-sectional qualitative; Thematic analysis | 6 adult NHL patients at least 18 months post-diagnosis who completed their treatment regimes. Age not indicated. All participants were over 18 years old, were either married or had a long-term partner, and received chemotherapy treatment although one participant started with “watch and wait”. Majority of participants were male (83%). Race/ethnicity not reported. | Focus groups (n=3) consisting of 2 participants each were conducted - prompted discussion about types of needs experienced throughout cancer experience and to gain level of understanding of why some needs were highlighted as important while others deemed not relevant | HCP communication (delivery of diagnosis important, clear delivery of individually tailored information important in feeling supported; sudden drop of this support after treatment created feelings of isolation and uncertainty; busyness of clinical environment encouraged ppl to suppress their distress/unmet needs)  Social support highly valued and was felt keenly if absent; disease characteristic (lack of outward signs, not understood well by GPs and friends/family, thought to be different from other cancers); peer support may not be easily accessible (e.g., transport/distance), nor helpful as seeing others becoming unwell makes it difficult to remain positive about own prognosis.  Social comparisons helped participants to understand situation and determine how well they were coping, but although seeing others thrive was source of hope, seeing others struggle was difficult and guilt may be evoked when others were perceived to be coping with worse situations.  Perceived sense of control - may differ between individuals - some felt taking control was empowering and enabled participants to own the experience and make sense of what was happening, whereas for others, surrendering control to clinicians was helpful |
| 11. | Wall et al., 2011 (UK) [42] | To identify and describe the essences of patients' experience during the period leading up to the diagnosis of NHL | Qualitative; phenomenological approach | 31 NHL patients who had been diagnosed for ≥ 2 months Mean age of 56 years, ranging 29-79 years. Race/ethnicity not reported. | Individual in-depth interviews lasting around 45-60 mins - participants asked to reflect on the period when they first became aware of a problem with their health, and to describe in depth what it was like for them during the period leading up to the diagnosis. | Factors highlighted:   1. Type of symptoms (i.e., sudden/acute or long-term and vague) 2. Healthcare professionals' communication   Information needs (i.e., uncertainty in what their symptoms mean, actively seeking out more information) |

*Note*: Summary of 11 qualitative studies reviewed, ordered alphabetically. Abbreviations: SD = standard deviation, HL = Hodgkin Lymphoma, NHL = Non-Hodgkin Lymphoma.

**Table 3**

*Summary of Mixed-Method Studies*

| 1. | Latella et al., 2020 (USA) [43] | To examine the frequency and causes/main sources of fear of cancer recurrence (FCR), how survivors cope with their FCR, and how oncologists have been helpful in reducing lymphoma survivors' FCR. | Cross-sectional mixed-methods; COPE Inventory, Thematic coding, Chi-square analyses, Fisher's exact test (follow-up differences) | 141 HL and DLBCL patients who were treated with curative intent, had imaging results indicative of being in remission, had completed chemotherapy (6 months post-treatment). All participants were sourced from a parent trial study [44]. Mean age = 46.08 years, range = 20-87 years. Majority were non-Hispanic White (56.7%). No additional information on sociodemographic and clinical characteristics. | 22-item Leventhal's Qualitative Interview questionnaire whereby FCR was operationalised as “worry” (e.g., how often participants worry about lymphoma coming back) | | **Two sources of FCR highlighted by some participants (n = 59)**   - Medical appointments (59%), whereby participants who endorsed situation-specific worry were more likely to report this as the main cause of their FCR - Aspects associated with relapse or secondary cancers (48%), whereby participants who endorsed intermittent worry were more likely to report this as the main cause of their FCR   **Participants (n = 116) used different coping strategies and these did not differ by the frequency of FCR endorsed.** Majority of participants used a self-sufficient approach (84.5%) which included active coping, religious and spiritual coping, acceptance, positive reinterpretation, planning, and restraint coping. 36.2% of participants used mental disengagement (avoidant oriented coping). 25.9% of participants used a socially supported approach including venting emotions and relying on emotional and instrumental social support.  **Several features of oncologists reduced FCR for participants (n = 102) and these features described did not differ by frequency of FCR.** Specific statements that oncologists say, such as statements about *low recurrence rates* (e.g., citing specific statistics, highlighting curability of disease, citing specific timeline milestones) and recommendations of *specific action plans* (e.g., “maintain a diet”, “follow up in 3 months”) were highlighted as helpful in reducing FCR. Moreover, oncologists’ positive attitude, honesty, and expertise instilled *trust in physician*. Their *communication of treatment progress* (e.g., praising test results, reassuring patients about effectiveness of treatment) and reassurance of *continuity of care* were also helpful. |
| --- | --- | --- | --- | --- | --- | --- | --- |
| 2. | Thompson et al., 2010 (USA) [45] | To assess anxiety and psychological impact of routine surveillance scans in long-term, asymptomatic survivors of adult aggressive lymphoma | Cross-sectional mixed methods: correlational and qualitative; Linear regression and grounded theory approach | 70 long-term curable aggressive lymphoma adult survivors > 2 years post-treatment (median of 4.9 years post-diagnosis). Median age of 47 years, ranging 23-80 years. Majority were female (64%), Caucasian (83%), graduated from college (53%), married (53%) and have children (54%). | Demographic, socioeconomic, and clinical data: self-reported (interviews). Medical records abstracted for verification.  Perceived relationship with physician: Patient-Physician Relationship Scale from NCI monograph on patient-centred communication in cancer care | Anxiety symptoms: STAI. STAI score ≥ 40 = clinically significant anxiety (i.e., anxiety caseness)  Individual qualitative interviews consisting of the following questions: “Tell me what it is like for you to have a follow-up CT scans", "How does it affect you emotionally", "What downsides are there for you". Only first 30 were transcribed and analysed. | Qualitative interviews revealed that many patients experienced fear of recurrence despite its low probability, with this peaking before receiving results, and dropping substantially following doctor's visit. Some suggested that results do provide reassurance that disease in remission, but experience is difficult. Scans were also suggested to be a reminder of the cancer.  Many demographic and clinical characteristics were not found to significantly associate with anxiety - examined age, employment status, having children, income, marital status, race, education, radiation treatment, number of treatment regimens, histological diagnosis, years since diagnosis. Females tended to have higher mean anxiety score than men, but this relationship was only approaching significance (p = .06).  In multivariate regression analysis, doctor-patient relationship and history of relapse remained significant predictors of anxiety. Particularly, worse doctor-patient relationship and history of relapse were associated with greater anxiety symptoms. |

*Note*: Summary of 2 mixed-methods studies reviewed, ordered alphabetically. Abbreviations: SD = standard deviation, HL = Hodgkin Lymphoma, NHL = Non-Hodgkin Lymphoma, DLBCL = Diffuse large B cell lymphoma, COPE = Creativity, Optimism, Planning and Expert Information, STAI = State-Trait Anxiety Index, NCI = National Cancer Institute.

**References**

1. Bellizzi, K.M., et al., *Positive and negative life changes experienced by survivors of non-Hodgkin's lymphoma.* Ann Behav Med, 2007. **34**(2): p. 188-99.

2. Cameron, C.L., et al., *Persistent symptoms among survivors of Hodgkin's disease: an explanatory model based on classical conditioning.* Health Psychol, 2001. **20**(1): p. 71-5.

3. El-Banna, M.M., et al., *Fatigue and depression in patients with lymphoma undergoing autologous peripheral blood stem cell transplantation.* Oncol Nurs Forum, 2004. **31**(5): p. 937-44.

4. Geffen, D.B., et al., *Post-traumatic stress disorder and quality of life in long-term survivors of Hodgkin's disease and non-Hodgkin's lymphoma in Israel.* Leuk Lymphoma, 2003. **44**(11): p. 1925-9.

5. Gil-Fernández, J., et al., *Quality of life and psychological well-being in Spanish long-term survivors of Hodgkin's disease: results of a controlled pilot study.* Ann Hematol, 2003. **82**(1): p. 14-8.

6. Hamilton, A.S., et al., *Predictors of use of complementary and alternative medicine by non-hodgkin lymphoma survivors and relationship to quality of life.* Integr Cancer Ther, 2013. **12**(3): p. 225-35.

7. Arora, N.K., et al., *Population-based survivorship research using cancer registries: a study of non-Hodgkin's lymphoma survivors.* J Cancer Surviv, 2007. **1**(1): p. 49-63.

8. Hammersen, F., et al., *Sleep quality and health-related quality of life among long-term survivors of (non-) Hodgkin lymphoma in Germany.* PLoS One, 2017. **12**(11): p. e0187673.

9. Husson, O., et al., *Satisfaction with information provision is associated with baseline but not with follow-up quality of life among lymphoma patients: Results from the PROFILES registry.* Acta Oncol, 2014. **53**(7): p. 917-26.

10. Loge, J.H., et al., *Psychological distress after cancer cure: a survey of 459 Hodgkin's disease survivors.* Br J Cancer, 1997. **76**(6): p. 791-6.

11. Magyari, F., et al., *Employment status and health related quality of life among Hodgkin-lymphoma survivors'- results based on data from a major treatment center in Hungary.* Health Qual Life Outcomes, 2017. **15**(1): p. 180.

12. Mariegaard, J., et al., *Prevalence of cognitive impairment and its relation to mental health in Danish lymphoma survivors.* Support Care Cancer, 2021. **29**(6): p. 3319-3328.

13. Matasar, M.J., et al., *Late morbidity and mortality in patients with Hodgkin's lymphoma treated during adulthood.* J Natl Cancer Inst, 2015. **107**(4).

14. Oerlemans, S., et al., *The course of anxiety and depression for patients with Hodgkin's lymphoma or diffuse large B cell lymphoma: a longitudinal study of the PROFILES registry.* J Cancer Surviv, 2014. **8**(4): p. 555-64.

15. Øvlisen, A.K., et al., *Mental health among patients with non-Hodgkin lymphoma: A Danish nationwide study of psychotropic drug use in 8750 patients and 43 750 matched comparators.* Am J Hematol, 2022. **97**(6): p. 749-761.

16. Øvlisen, A.K., et al., *Depression and anxiety in Hodgkin lymphoma patients: A Danish nationwide cohort study of 945 patients.* Cancer Med, 2020. **9**(12): p. 4395-4404.

17. Pettengell, R., et al., *The impact of follicular lymphoma on health-related quality of life.* Ann Oncol, 2008. **19**(3): p. 570-6.

18. Poe, J.K., J.W. Hayslip, and J.L. Studts, *Decision making and distress among individuals diagnosed with follicular lymphoma.* J Psychosoc Oncol, 2012. **30**(4): p. 426-45.

19. Posluszny, D.M., et al., *Existential challenges experienced by lymphoma survivors: Results from the 2010 LIVESTRONG Survey.* J Health Psychol, 2016. **21**(10): p. 2357-66.

20. Roper, K., et al., *Health-related quality of life after treatment of Hodgkin lymphoma in young adults.* Oncol Nurs Forum, 2013. **40**(4): p. 349-60.

21. Smith, S.K., et al., *Post-traumatic stress outcomes in non-Hodgkin's lymphoma survivors.* J Clin Oncol, 2008. **26**(6): p. 934-41.

22. Smith, S.K., et al., *The impact of cancer and quality of life for post-treatment non-Hodgkin lymphoma survivors.* Psychooncology, 2010. **19**(12): p. 1259-67.

23. Soares, A., et al., *Association of social network and social support with health-related quality of life and fatigue in long-term survivors of Hodgkin lymphoma.* Support Care Cancer, 2013. **21**(8): p. 2153-9.

24. Spector, D.J., et al., *Are lifestyle behavioral factors associated with health-related quality of life in long-term survivors of non-Hodgkin lymphoma?* Cancer, 2015. **121**(18): p. 3343-51.

25. Smith, S.K., et al., *Quality of life among long-term survivors of non-Hodgkin lymphoma: a follow-up study.* J Clin Oncol, 2013. **31**(2): p. 272-9.

26. Tilch, M.K., et al., *Burden of depression and anxiety disorders per disease codes in patients with lymphoma in Germany.* Support Care Cancer, 2022. **30**(3): p. 2387-2395.

27. Troy, J.D., et al., *Patient-reported distress in Hodgkin lymphoma across the survivorship continuum.* Support Care Cancer, 2019. **27**(7): p. 2453-2462.

28. Varela, V.S., et al., *Posttraumatic stress disorder (PTSD) in survivors of Hodgkin's lymphoma: prevalence of PTSD and partial PTSD compared with sibling controls.* Psychooncology, 2013. **22**(2): p. 434-40.

29. Ng, A.K., et al., *A comparison between long-term survivors of Hodgkin's disease and their siblings on fatigue level and factors predicting for increased fatigue.* Ann Oncol, 2005. **16**(12): p. 1949-55.

30. Walburg, V., et al., *Fear of cancer recurrence in Non- and Hodgkin lymphoma survivors during their first three years of survivorship among French patients.* Psychol Health Med, 2019. **24**(7): p. 781-787.

31. Wang, Y., et al., *Measurement of distress in Chinese inpatients with lymphoma.* Psychooncology, 2013. **22**(7): p. 1581-6.

32. Chircop, D. and J. Scerri, *The use of metaphors in non-Hodgkin's lymphoma patients undergoing chemotherapy.* J Adv Nurs, 2018. **74**(11): p. 2622-2629.

33. Friesen-Storms, J., et al., *Stories of Lymphoma Survivors in Early Aftercare: A Narrative Inquiry.* Cancer Nurs, 2021. **44**(6): p. 489-498.

34. Glasdam, S., C. Bjerström, and C. Engberg de Carvalho, *Coping strategies among patients with malignant lymphoma- a qualitative study from the perspectives of Swedish patients.* Eur J Oncol Nurs, 2020. **44**: p. 101693.

35. Hackett, F. and M. Dowling, *Lymphoma survivors' experiences at the end of treatment.* J Clin Nurs, 2019. **28**(3-4): p. 400-409.

36. Johansson, E., et al., *Symptoms before, during, and 14 months after the beginning of treatment as perceived by patients with lymphoma.* Oncol Nurs Forum, 2010. **37**(2): p. E105-13.

37. Matheson, L., et al., *The Experiences of Young Adults With Hodgkin Lymphoma Transitioning to Survivorship: A Grounded Theory Study.* Oncol Nurs Forum, 2016. **43**(5): p. E195-e2014.

38. Monterosso, L., et al., *A qualitative study of the post-treatment experiences and support needs of survivors of lymphoma.* Eur J Oncol Nurs, 2017. **28**: p. 62-68.

39. Payne, J.B., et al., *Patient and caregiver perceptions of lymphoma care and research opportunities: A qualitative study.* Cancer, 2019. **125**(22): p. 4096-4104.

40. Ruan, J., et al., *The Illness Experiences of Chinese Patients Living With Lymphoma: A Qualitative Study.* Cancer Nurs, 2020. **43**(4): p. E229-e238.

41. Swash, B., N. Hulbert-Williams, and R. Bramwell, *'Haematological cancers, they're a funny bunch': A qualitative study of non-Hodgkin's lymphoma patient experiences of unmet supportive care needs.* J Health Psychol, 2018. **23**(11): p. 1464-1475.

42. Wall, C., S. Glenn, and H. Poole, *Experiences prior to diagnosis of non-Hodgkin lymphoma: a phenomenological study.* J Adv Nurs, 2011. **67**(11): p. 2363-72.

43. Latella, L.E., et al., *Fear of cancer recurrence in lymphoma survivors: A descriptive study.* J Psychosoc Oncol, 2020. **38**(3): p. 251-271.

44. Parker, P.A., et al., *Protocol for a cluster randomised trial of a communication skills intervention for physicians to facilitate survivorship transition in patients with lymphoma.* BMJ Open, 2016. **6**(6): p. e011581.

45. Thompson, C.A., et al., *Surveillance CT scans are a source of anxiety and fear of recurrence in long-term lymphoma survivors.* Ann Oncol, 2010. **21**(11): p. 2262-2266.
